# Comparative analysis of intrinsic skin aging between Caucasian and Asian subjects by slide‐free in vivo harmonic generation microscopy

**DOI:** 10.1002/jbio.201960063

**Published:** 2019-12-05

**Authors:** Kuan‐Hung Lin, Yi‐Hua Liao, Ming‐Liang Wei, Chi‐Kuang Sun

**Affiliations:** ^1^ Graduate Institute of Biomedical Electronics and Bioinformatics National Taiwan University Taipei Taiwan; ^2^ Department of Dermatology National Taiwan University Hospital and National Taiwan University College of Medicine Taipei Taiwan; ^3^ Graduate Institute of Photonics and Optoelectronics and Department of Electrical Engineering National Taiwan University Taipei Taiwan

**Keywords:** Asian, Caucasian, dermal papillae, harmonic generation microscopy, in vivo imaging, intrinsic aging, race, viable epidermis

## Abstract

Phenotypical and functional differences in the intrinsic skin aging process of individuals between Caucasians and Asians have generated considerable interest in dermatology and cosmetic industry. Most of the studies focused on the stratum corneum, and in some other studies inter‐individual differences overwhelms the racial difference. None of the studies comparatively analyzes the difference from the histopathological point of view. Here we report our harmonic generation microscopy study to analyze the difference of intrinsic aging between Caucasian and Asian skin from a histopathological point of view. As a result, the cellular and nuclear areas of basal cells in Caucasian subjects were found to increase at the same rate as the Asian subjects, ideal for scoring age. The maximum thickness of the viable epidermis, the dermal papilla (DP) volume per unit area and the depth of the DP zone in Caucasians were found to decrease at faster rates than those in Asians.
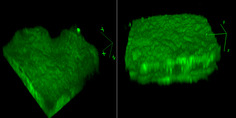

## Graphical abstract

Phenotypical and functional differences in the intrinsic skin aging process of individuals between Caucasians and Asians have generated considerable interest in dermatology and cosmetic industry. Most of the studies focused on the stratum corneum, and in some other studies inter‐individual differences overwhelms the racial difference. Here we report our harmonic generation microscopy study to analyse the difference of intrinsic aging between Caucasian and Asian skin from a histopathological point of view.

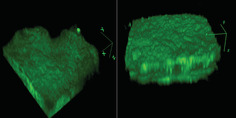

## 1 INTRODUCTION

Aging is an inalterable process in which both extrinsic and intrinsic determinants induce a loss of structural integrity and physiological function 1. Extrinsic skin aging is caused by exogenous factors such as sun exposure, environmental toxins and infectious agents 2, whereas intrinsic skin aging is determined by genetic influences, age and internal factors such as hormones and metabolic substances 3. Histologically, the intrinsic aged skin shows a thinning of all layers, epidermal and dermal atrophy, increased heterogeneity in size of basal cells, flattening of the dermoepidermal junction, decreased vasculature, as well as reduced numbers of melanocytes, Langerhans cells and fibroblasts 4. The increased basal cell size induces an increased intercellular space, which would reduce the barrier function; and further, accelerates the aging process. Due to the reduced dermoepidermal contact area while the stiffness increases, aging leads to a more rigid dermis which decreases the ability of the skin to resist shear forces 5. Therefore, the skin appears roughened and deeply wrinkled. These intrinsic aging‐related changes induce increased vulnerability of skin as well as the disturbed skin barrier function, which cause increased incidence of inflammatory or infectious skin disorders and lead to an aged appearance.

Phenotypical and functional differences in the skin of individuals between Caucasians and Asians have generated considerable interest in dermatology and cosmetic industry. In previous studies, some structural and functional differences between racial skins were observed. In stratum corneum, the number of cell layers is higher in Caucasians 6 and the water content is higher in Asians 7. In dermis, the dermal thickness, collagen content and melanin content are higher in Asians 8. On the other hand, there were no racial differences observed in skin elasticity on the volar forearm by using the Twistometer 9. Moreover, the different effect of aging between racial skins has further generated interest in how the racial factor has contributed to this. A number of studies show some racial difference in the aging of skin that Caucasian skin wrinkles and sags at a much faster rate when compared with Asian skin 10, 11. However, none of them comparatively analyzes the difference of intrinsic aging between Caucasian and Asian skin from the histopathological point of view.

Skin biopsy is a medical test commonly performed by dermatologists involving the extraction of tissues from a living subject for pathological examination 12. However, its invasive nature makes it not a suitable way to investigate skin aging. The in vivo harmonic generation microscopy could be a unique tool for investigating the cytological changes of intrinsic skin aging due to its combined capability of noninvasive label‐free slide‐free imaging with high 3D spatial resolution and high penetrability. In our previous study, we quantitatively analyzed changes in keratinocytes related to chronological skin aging by in vivo harmonic generation microscopy (HGM) and found that the cellular and nuclear size of basal keratinocytes was significantly increased with advancing age in Asian skin 13. In our another study of dermal papillae (DP) in Asian skin, we found the 3D interdigitation index decreased with age, while dermal DP volume, and the collagen density in DP remained constant over time 14. The aim of this study is to investigate if these cytological and tissue parameters of intrinsic skin aging differ with Caucasian skin.

In our comparative analysis between the Caucasians and Asians, the cellular and nuclear areas of the basal cells in Caucasian subjects were found to increase at the same rate as the Asian subjects. Our study concludes that the primary factor causing a different aging outlook between the Caucasian and Asians is the dermal papillae zone (DPZ)‐related parameters while our skin's intrinsic age among two racial groups can be evaluated using the cellular and nuclear areas of the basal cells as scoring indices.

## 2 METHODS

### 2.1 Study population

A total of 31 Caucasian subjects, including five females and seven males aged 19 to 29 years, five females and eight males aged 30 to 59 years and two females and four males aged 60 to 79 years, were investigated. None of the subjects had skin disease in the imaged areas. We used two different microscope systems in this study; one microscope system was used for investigating 23 Caucasian subjects, and the other was used for investigating eight Caucasian subjects. These two microscopes have been calibrated to reduce the intensity or scale difference caused by factors such as laser intensity and resolution of the *Z* stage. Our studied Asiatic population 13, 14 was Han Chinese, which can be a representative group for Asian because it has about a 1.16 billion population accounting for 19% of the world's total population. This study was conducted in accordance with the principles of the Declaration of Helsinki, and the study protocol was approved by the Research Ethics Committee of National Taiwan University Hospital (under IRB No. 200903064D, NTUH‐REC). Informed consent was obtained from each subject prior to study entry.

### 2.2 Harmonic generation microscope system

**Figure 1:**
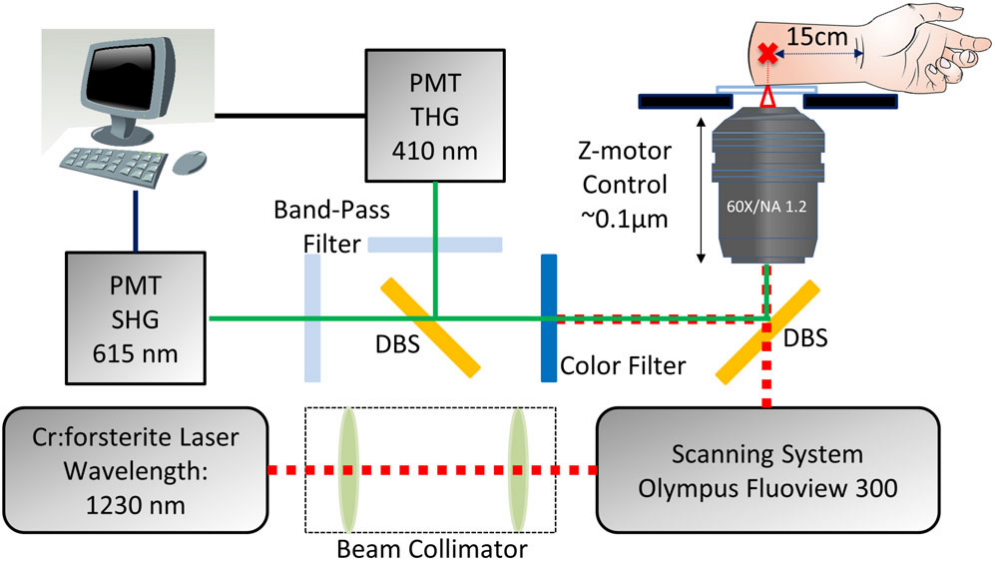
A schematic showing the 1230‐nm‐based inverted harmonic generation microscope system adapted from a commercial laser scanning system from Olympus combined with an inverted microscope; PMT: photomultiplier tube; DBS: dichroic beam splitter 16

**Figure 2:**
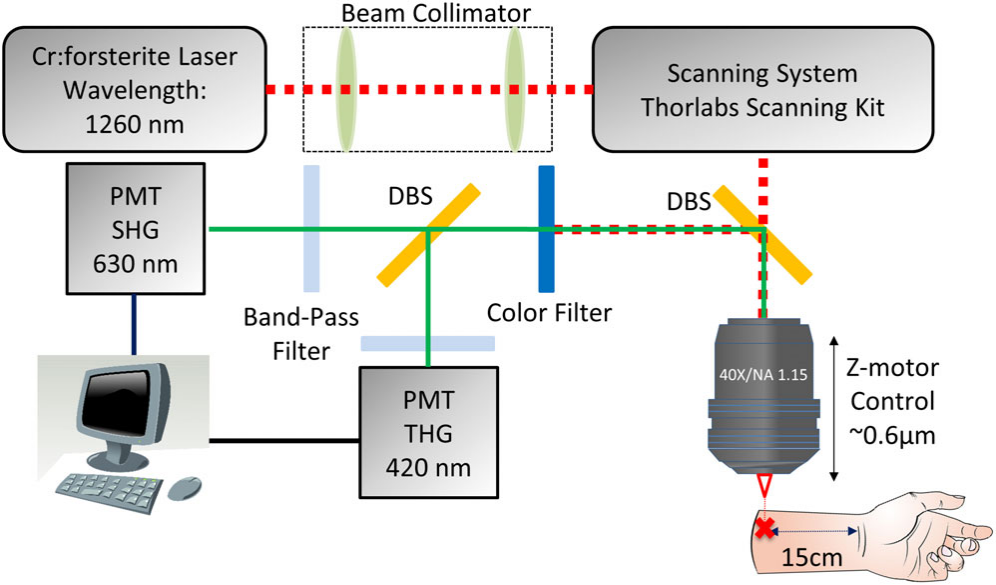
A schematic showing the 1260‐nm‐based upright harmonic generation microscope system adapted from a commercial laser scanning system from Thorlabs combined with an upright microscope 17; PMT: photomultiplier tube; DBS: dichroic beam splitter

A 1230‐1260‐nm femtosecond chromium‐forsterite laser was used for excitation to lessen skin attenuation from scattering and absorption of the human skin, and the second‐ and third‐harmonic phenomena were generated simultaneously. One HGM system as previously described 15 was adapted from a commercial confocal scanning system (FV300, Olympus, Tokyo, Japan) combined with an inverted microscope (IX71, Olympus, Tokyo, Japan). In this study, a 60× water‐immersion objective (Olympus, UplanApo/IR) with NA 1.2 was used, and two photomultiplier tubes (PMTs) backward‐collected the second‐ and third‐harmonic generation (SHG and THG) signals (Figure 1). The volar of the forearm is a representative photoprotective area to study for intrinsic aging in the Dermatology field. Our imaging system can be applied in other photoprotective body areas such as buttocks, but it is not very convenient for the subjects. In this system, subjects had to place their forearm on the objectives with the ventral side downward, and the imaging depth was controlled by the motor of *z* direction with a 0.1‐μm resolution. A submicron and 2‐μm resolution in the lateral and axial directions and a greater than 300‐μm penetrability were achieved 16. The second HGM system 17 was adapted from a galvo‐resonant scanning head (Thorlabs Laser Scanning Essentials Kit, Thorlabs, New Jersey). The scanning pattern was focused onto human skin by a 40× water immersion objective (Olympus, UAPON 40XW340) with a NA of 1.15, and two PMTs backward‐collected the second‐ and third‐harmonic generation signals (Figure 2). In this second system, subjects have to place their forearm under the objectives with the ventral side upward, and the imaging depth was controlled by the motor of *z* direction on the objective with a 0.6‐μm resolution. A 363 nm and 1.8‐μm resolution in the lateral and axial directions and a higher than 300‐μm penetrability were achieved.

### 2.3 Imaging procedure

**Figure 3:**
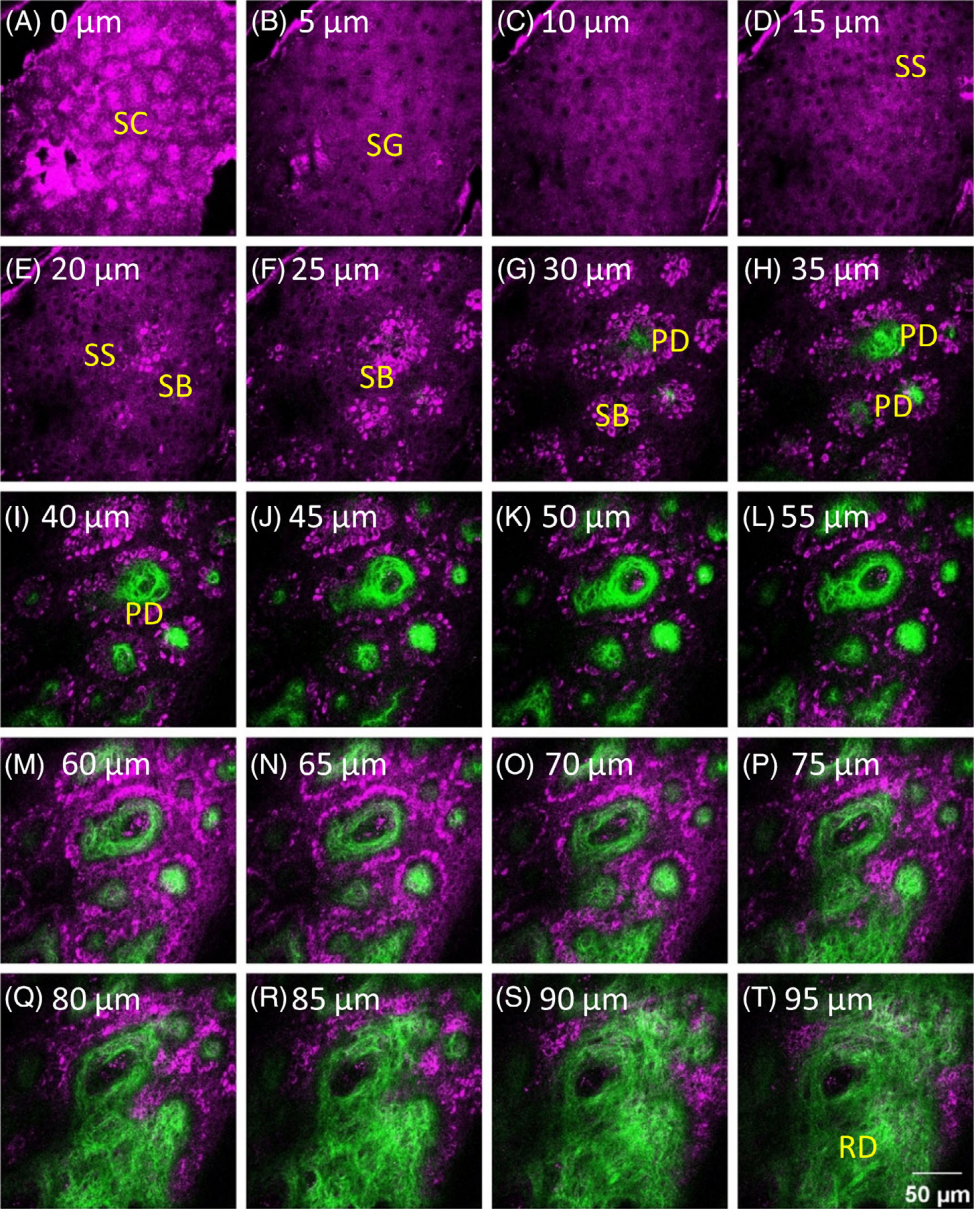
A part of a representative XYZ image stack combining SHG and THG signals obtained from a 43‐year‐old male subject. The image stack captured different skin layers, including the stratum corneum (SC), the stratum granulosum (SG), the stratum spinosum (SS), the stratum basale (SB), the papillary dermis (PD) and the upper reticular dermis (RD). The depths of images are labeled, and the depth of the first frame of the stack that showed the stratum corneum was set as 0 μm. SHG and THG signals are represented by pseudo‐colors, green and purple. Image dimensions are 235.7 × 235.7 μm

The HGM images of the left ventral forearm were obtained at about 15 cm proximal to the subject's wrist. Through the XYZ mode of the in vivo HGM system, the objective could be moved in the z direction with a set step size, and the image stacks were obtained with a step size of 1.8, 2 or 5 μm. The XYZ image stacks of the skin were obtained beginning from the stratum corneum, through the stratum granulosum, the stratum spinosum, the stratum basale, the papillary dermis, to the upper reticular dermis (Figure 3). During the imaging process, the voltages of the two PMTs were fixed independent of the imaging depth to maintain the second harmonic generation (SHG) and third harmonic generation (THG) signals in equal brightness for the accuracy of quantitative analysis. Backward SHG and THG signals were collected to form 512 × 512 pixel images at two frames per second, with a 235.7 × 235.7 μm or 232.7× 235.7 μm field of view (FOV). Two to seven image stacks were collected for each subject at different locations. A total of 150 XYZ image stacks, including 12 stacks with a step size of 2 μm, 27 stacks with a step size of 1.8 μm and 138 stacks with a step size of 5 μm, were analyzed. The maximum, the minimum and the average numbers of the image stacks obtained from one subject were 7, 2 and 4.1, respectively. The experimental skin areas, with multiple locations, were exposed to the laser for no more than 30 minutes in one subject. In one area, the total laser exposure time was less than 3 minutes, with an accumulated energy no more than 18 J. The power of the laser measured right after the objective was limited within 100 mW, and the total energy projected on the different locations of the skin was no more than 180 J. At the end of the experiment, the inspected skin area was immediately examined by taking photographs. The entire process took about 1 or 2 h, including the explanation of the experiment to the subject, the laser exposure, and the evaluation of health condition. The subjects were informed to feel free to contact the laboratory technicians/research nurse if they felt any discomfort during or after the experiment. No short‐term side effects, including inflamed skin, scrape, scald, were observed. The subjects stated that the procedure was comfortable and caused neither itch nor pain. No subject felt any heat on the 100 mW Cr:F laser‐irradiated skin.

A previous study indicated a THG efficiency of 1.4 × 10^−9^ in biotissues 18 by using near infrared (NIR) femtosecond light. Thus generated THG at 420 nm will be on the level of 0.14 nW, and was too weak to generate any long term damage effect on human skin. For multiphoton ionization effect with tightly focused NIR femtosecond light under a high NA objective, live embryo cells irradiated with 730 to 800 nm (1.7‐1.55 eV) beams of >1 mW average power (total exposure = 0.2 J) were found to inhibit cloning efficiency 19, indicating DNA damage. A skin damage study with 750 nm tightly focused femtosecond light 20 indicated the same DNA damage but with a higher dosage allowance (10 scans, 30 mW, 32 s per scan; total exposure 9.6 J) in human skin. When the excitation photon energy is lowered toward longer wavelength, the order number of nonlinear damage process increases, thus drastically increasing the nonlinear damage threshold 21. For 1030 nm light, it will require four‐photon processes to generate the similar effect of 5 eV photon light. For 1260 nm (0.98 eV) light, the nonlinear process required to generate similar 5 eV effects will be a five‐photon process, a less likely event under low pulse energy on the order of or less than 1 nJ, which is our case. By shifting the femtosecond laser wavelength even further to 1230 nm, with 120 mW average power (3 minutes continuously per embryo, total exposure = 21.6 J), the HGM‐imaged embryos under tightly focused light were found to maintain full viability 15. The blastocyst development rate 15 (total mouse embryos n = 165) of the HGM‐imaged embryos with laser exposure is the same as nonimaged embryos, thus indicating no long‐term effect on DNA damage or cloning efficiency. This conclusion was supported by other 1230 nm‐based HGM studies of in vivo zebrafish embryos 22, 23 developments (total exposure 7200 J per embryo), and an in vivo hamster buccal tissue study 24 (1620 J total exposure) with immediate histological examinations on irradiated tissues. With average power and imaging time (or dosage) both lower than the previous embryo studies but with a longer excitation wavelength, we thus do not expect any long‐term damage on human skin through multiphoton ionization effect with HGM.

### 2.4 Analysis protocols

The image stacks were analyzed to understand the morphological features of the viable epidermis (VE) and the dermal papillae. For comparative analysis, the analysis thickness of viable epidermis, the cellular, nuclear size and NC ratio of granular cells, and the depth of DPZ were manually analyzed with the same protocols as in our previous study on Asian skin 14. However, the cellular, nuclear size and NC ratio of basal cells, the DP volume per unit area, the DP volume ratio and the 3D interdigitation index were analyzed by using the modified codes developed based on previously published algorithms 14. These parameters were manually analyzed in our previous study on Asian skin. Moreover, the 2D collagen density in DP and the collagen volume in DP per unit area were analyzed by a modified algorithm from our previous algorithm 14. During comparison, 2D collagen density in DP and the collagen volume in DP per unit area of Asian skin were reanalyzed by using the new algorithm.

#### 2.4.1 In vivo analysis of viable epidermis

The viable epidermis lies beneath the stratum corneum, which includes the stratum granulosum, the stratum spinosum and the stratum basale, where nucleated keratinocytes could be observed. For example, in Figure 3A, no cells with nuclei could be observed in the stratum corneum, and the frame showing cells with nuclei first appeared in the stratum granulosum in Figure 3B and it was defined as the topmost layer of the viable epidermis.

##### *Viable epidermis thickness*

The thickness of the viable epidermis (*T*_VE_) was measured from the stratum granulosum to the stratum basale. However, the interface between the epidermis and the dermis is a wave‐like structure, and whether rete ridges, which are the downward projections of the epidermis between the dermal papillae, should be included in the thickness measurement was an issue. Therefore, measurements both including and excluding the depth of rete ridges, which were defined as the minimum and the maximum thickness of the viable epidermis, respectively, were performed. For example, in Figure 3, the minimum thickness of the viable epidermis (*T*_VEm_) was the step size multiplied by the count of frames from (b) to (f), and the maximum thickness of the viable epidermis (*T*_VEM_) was the step size multiplied by the count of frames from (b) to (q). Both thicknesses were measured for every image stacks, and the average and standard deviation (SD) of the thickness of each subject were calculated based on the respective thicknesses of the image stacks of all subjects (Table 1).

**Table 1:** Summary of parameter definitions and notations in the in vivo analysis of the viable epidermis

Parameter	Definition	Notation
Minimum thickness of viable epidermis	Thickness of the cellular epidermis determined by the top of the uppermost papillae	*T*_VEm_
Maximum thickness of viable epidermis	Thickness of the cellular epidermis determined by the valleys of the papillae	*T*_VEM_
Average area of granular cell 13	Average area of granular cell under the *en face* view	*A*_G_
Average area of granular nucleus 13	Average area of granular nucleus under the *en face* view	*A*_GN_
NC ratio of granular cell 13	$\frac{\text{Total nucleus area of granular cell}\;}{\text{Total cytoplasm area of granular cell}}$	NC_G_
Average area of basal cell 13	Average area of basal cell under the *en face* view	*A*_B_
Average area of basal nucleus 13	Average area of basal nucleus under the *en face* view	*A*_BN_
NC ratio of basal cell 13	$\frac{\text{Total nucleus area of basal cell}}{\text{Total cytoplasm area of basal cell}}$	NC_B_

“ImageJ” was used to open “.tiff” format of image stacks, and Excel was used to record the number of the first frames showing the SG, the number of the first frames showing the PD, and the number of the last frames showing the SB. The XYT mode image stacks, the stacks in which typical granular cells could not be recognized, and the stacks in which the bottom of the stratum basal could not be observed were excluded.

##### *Cell size and NC ratio of granular cells*

Since the THG signals in the cytoplasm of granular cells are enhanced by the keratohyaline granules, the nucleus and cytoplasm areas of the granular cells can be observed with dark and bright THG contrasts. SG can be differentiated from SS using THG. SG is characterized by its large cell size (around 500‐600 μm^2^) 13 and course keratohyaline granules in the cytoplasm. “ImageJ” was used to open “.tiff” format of image stacks, and Excel was used to record the cellular and nuclear size of granular cells. The nucleus‐cytoplasm ratio (NC ratio) is the ratio of the size of the nucleus area under the *en face* view to the size of the cytoplasm area under the *en face* view. An increase in NC ratio indicates cellular atypia, which might be a signature of premalignant or malignant tumor 25. Only one or two THG images obtained at the stratum granulosum were used to calculate the average NC ratio due to the thin overall thickness of the stratum granulosum. In each THG image, the total areas of both nuclei and cytoplasm were obtained to calculate the NC ratio. For analyzing the cellular and nuclear size of granular cells, at least two images (per subject) containing the majority of granular cell sections were chosen. In each image, all the cells were selected for measuring the cell and nucleus areas and at least 25 cells were selected for each subject. These criteria were applied to avoid measuring an off‐centered cross‐sectional area of the cell and the nucleus. For each chosen THG image, the total areas of both the nuclei and cytoplasm of the granular cells were manually circled using the “ROI manager” in ImageJ. “Image → color → split channels” was used to observe the THG image and to adjust the “Contrast” from 0‐254 to 50‐204. The stacks in which granular cells could not be observed were excluded for area measurement. To avoid the difference contributed from different personal standards, observers were trained to make sure that they share the same analysis standards so that the analyzed results will be well aligned.

##### *Cell size and NC ratio of basal cells*

For analyzing the cellular and nuclear size of basal cells, at least three images (per subject) containing the majority of basal cell sections with nuclei were randomly chosen. In each image, the first 5 to 10 cells with the largest cross‐sectional area were selected 26 for measuring the cell and nucleus areas to ensure that the central region of the cross‐section was chosen, and at least 25 cells were selected for each subject. For the NC ratio analysis of basal cells, at least two images (per subject) containing the majority of cell sections were chosen. In each image, all the sectioned cells with nuclei were selected, and the total cytoplasmic and nuclear area was obtained by summing up the individual cytoplasmic and nuclear area. The volumetric NC ratio was obtained by dividing the total nuclear area by the total cytoplasmic area.

#### 2.4.2 In vivo analysis of dermal papilla zone

##### *Dermal papilla zone*

In the human skin, the epidermis and the dermis are joined at the interface, which is known as the dermal‐epidermal junction, and the dermal papilla zone (DPZ) is defined as the layer where the epidermis and the dermis coexist 14. Therefore, in the XYZ image stacks, the dermal papilla zone began when the dermis first appeared in the shallower layer and ended when the basal cells disappeared. The subsequent analysis was focused on the features of the dermal papillae within the dermal papilla zone.

##### *Depth of dermal papilla zone*

The number of frames between the first observation of DP and the last observation of SB was counted and multiplied with the imaging step size to estimate the depth of DPZ (*T*_DPZ_) 14. For each subject, the respective depth of the dermal papilla zone for each of his or her stack was obtained. Based on these depths, the depth for each subject was obtained from the average of stacks and the intra‐subject SD (standard deviation) was calculated.

##### *Digital image processing for volume per unit area, volume ratio and 3D interdigitation index*

**Figure 4:**
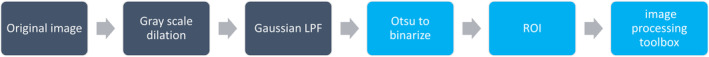
Algorithmic framework for analyzing the surface area and the volume of the dermal papilla zone. We segmented the region of DPZ as our region of interest (ROI)

In our previous study, the surface area and the volume of the dermal papilla zone were manually analyzed 14. In this study, we modified the previous algorithm for analyzing collagen fibers to segment the dermal papilla region. As shown in Figure 4, the processing route represents the algorithmic framework to define the DPZ as our region of interest (ROI), and the blue framework represents the part where we intend to edit the segment in the dermal papilla region 14. Because this algorithm segments the DPZ as the ROI 14 using the Otsu‐based method, the ROI of the DPZ can be used to calculate the surface area and the volume of the dermal papillae. The Otsu model adequately matches the intrinsic property of our data, which are composed of the following three categories of pixels: collagen, intensity‐saturated collagen and background noise. The Otsu's method was applied in the middle frame of an image stack to maintain the connectivity of the ROI, and the obtained threshold was set as the standard threshold for the image stack to perform the segmentation. To binarize the image, we defined the top two classes in the three‐class Otsu‐thresholded images as potential collagen areas and set them as 1. The total occupied section area and its circumference of the dermal papillae were calculated for each frame within the dermal papilla zone to calculate the following parameters. If a part of the circumference overlapped the border lines of the frame, the length of that part was excluded in the calculation. As it got deeper, the dermal papillae would occupy more area in the frame. Nevertheless, for most of the XYZ stacks, the occupied section area of the dermal papillae in the deepest frame was smaller than the whole frame area, which means that the bottom area of the dermal papilla zone need to be defined as the max occupied section area of the dermal papillae instead of the whole frame area. Within the dermal region, sweat glands appeared occasionally, of which the area should not be considered. Sweat glands were observed as large dark holes surrounded by THG signals of ductal cells in the dermis. The bottom area of the dermal papilla zone was defined as the maximum occupied section area (OA_max_) of different dermal papilla, excluding the area of sweat glands. OA_max_ was a normalization factor for comparing the physical properties of the DPZ across the image stacks.

The DP height was calculated by summing up the number of consecutive frames and multiplying by the step size. For the estimation of the surface area and the volume, the section areas and their circumferences were calculated in consecutive frames, and the volume and the surface area were estimated by the rectangular estimation, which was applied in our previous study 14. For the volume estimation, the calculation was carried out by summing up the section areas multiplied by the step size. Using the same principle, the surface area was calculated by adding all the products of the circumference and the step size, the area differences between the pairs of section areas in two successive frames, and the top section area.

For the estimations of the volume and the interface area of the dermal papillae, the tiny spaces between the collagen fibers and the capillaries inside the dermal papillae were all recognized as parts of dermal papillae. Using the estimated height, the interface area and the volume, the following parameters were defined and calculated using the image‐processing tool box in Matlab 2016: the dermal papilla volume per unit area, the dermal papilla volume ratio within the dermal papilla zone and the 3D interdigitation index.

##### *2D collagen density in DP and collagen volume in DP per unit area*

To study the distribution of imaged collagen fibers, the collagen area was segmented by using a modified algorithm from our previous one 14, 19. The 2D collagen density (D) was defined as the ratio between the area of collagen fibers and that of the DPZ. A higher area ratio is indicative of densely‐packed collagen fibers. In each XYZ image stack, the total 2D collagen density was calculated from the frame that dermis first appeared in the shallower layer and to the frame that the basal cells disappeared in the deeper layer. The collagen density within DPZ for a subject was calculated by the sum of the collagen area obtained from all his or her stacks divided by the sum of the dermal papillae area, with capillary area included. For a subject, the respective collagen density of every his or her stack was calculated, and the intra‐subject standard deviation was obtained between these collagen densities.

The collagen volume in DP per unit area (CV) of a subject, which revealed how much volume of collagen fibers spread on the skin, was calculated by the dermal papillae volume per unit area from all his or her image stacks multiplied by the 2D collagen density in DP. The collagen volume in DP per unit area of each stack was calculated, and the intra‐subject standard deviation for a subject was obtained between the respective results of his or her stacks.

We did not analyze the SAAID (SHG‐to‐two photon excited auto fluorescence aging index) 27 due to the simple fact that there is no detectable autofluorescence 15, 28, 29 from human skin under 1260 nm femtosecond excitation (Table 2).

**Table 2:** Summary of parameter definitions and notations in the in vivo analysis of dermal papillae within the dermal papilla zone

Parameter	Definition	Notation
Depth of DPZ 14	Depth of DPZ	T_DPZ_
DP volume per unit area 14	$\frac{\text{Total}\;\mathrm{DP}\;\text{volume}}{{\mathrm{OA}}_{\max}}$	*V*
DP volume ratio within DPZ 14	$\frac{\text{Total}\;\mathrm{DP}\;\text{volume}}{{\mathrm{OA}}_{\max}\times T_{\mathrm{DPZ}}}$	*R*
3D inter‐digitation index 14	$\frac{\text{Total}\;\mathrm{DP}\;\text{interface area}}{{\mathrm{OA}}_{\max}}$	*I*
2D collagen density in DP 14	$\frac{\text{Total collagen area in}\;\mathrm{DP}}{\text{Total}\;\mathrm{DP}\;\text{area}}$	*D*
Collagen volume in DP per unit area	DP volume per unit area × 2D collagen density in DP	CV

### 2.5 Statistical protocol

Statistical analysis was performed using IBM spss Statistics version 22. A set of parameters and their intra‐subject standard deviations were acquired for each subject using the abovementioned methods. Simple linear regression with age as a variable was used to investigate age‐related changes in the Caucasian subjects. In the analyses of covariance (ancova) reported in the results section, race (Caucasian vs Asian) was used as the blocking variable and age as a covariate (to control for the dependent variable). For statistical analysis, *P* values (denoted as *P*) were acquired for age and the interaction between age and race. This helps us to determine if age and race factors are independent of each other. For instance, they could be considered as independent if the effect of age on the cell size remains the same irrespective of whether we take race into consideration. In other words, if the interaction between age and race is significant, the aging trend between the Caucasians and the Asians is different. A *P* value of <.05 was considered to be statistically significant. If the interaction between age and race (denoted as age*race) is significant, the ancova model was not performed. If only one variable showed statistical significance, the correlation coefficient was acquired from the simple linear regression model. To analyze the differences among age groups, data were sorted out according to the age groups of 19 to 29, 30 to 59 and 60 to 79 years. One‐way analysis of variance (anova) or Kruskal‐Wallis test was used for comparisons among the three age groups in the Caucasian subjects. If anova showed significance for the age group factor, posthoc analysis was performed by the Fisher's Least Significant Difference (LSD) method to inspect difference of which two groups was significance. Furthermore, if Kruskal‐Wallis test showed significance for the age group factor, Mann‐Whitney U test was performed to inspect difference of which two groups was significance. If race showed statistical significance in the two‐way anova, the three age groups were further divided by race into six groups. The average and standard deviation for each group were calculated and presented as the average ± SD. Among six different groups, three different age groups with two different races, the differences in the average values of the dependent variable between the two racial groups were analyzed by the Mann‐Whitney U test.

## 3 RESULTS

### 3.1 In vivo analysis of viable epidermis for Caucasians

#### 3.1.1 Viable epidermis thickness

For the minimum thickness of viable epidermis (*T*_VEm_), simple linear regression showed no statistically significant correlation with age in Caucasian subject (Table 3; *P*_min_ for age in Caucasians = .94). The average minimum thickness of viable epidermis for age groups 19‐29, 30‐59 and 60‐79 years were 32.36 ± 14.78 μm, 33.52 ± 17.70 μm and 31.54 ± 5.71 μm, respectively, without statistically significant difference for Caucasian subjects (*P* = .82; Kruskal‐Wallis test). Therefore, the minimum thickness of viable epidermis did not correlate with age.

**Table 3:** Summary of the in vivo analysis of the viable epidermis and DPZ

Analysis summary		
	*P* value	
	Age (C)	Age*race
Minimum thickness of viable epidermis	NS	NS
Maximum thickness of viable epidermis	*P* < .05	*P* < .05
Average area of granular cell	NS	*P* < .05
Average area of granular nucleus	*P* < .05	*P* < .05
NC ratio of granular cell	NS	NS
Average area of basal cell	*P* < .001	NS
Average area of basal nucleus	*P* < .001	NS
NC ratio of basal cell	NS	NS
Depth of DPZ	*P* < .001	*P* < .01
DP volume per unit area	*P* < .01	*P* < .01
DP volume ratio within DPZ	*P* < .05	NS
3D interdigitation index	*P* < .01	NS
2D collagen density in DP	NS	NS
Collagen volume in DP per unit area	*P* < .01	NS

Note: Age (C) summarizes correlation with age in Caucasian subject based on linear regression analysis. *P* value for age (C) is calculated by anova while *P* value for age*race is calculated by ancova. The interaction between age and race is denoted as age*race.

Abbreviation: NS, not significant.

**Figure 5:**
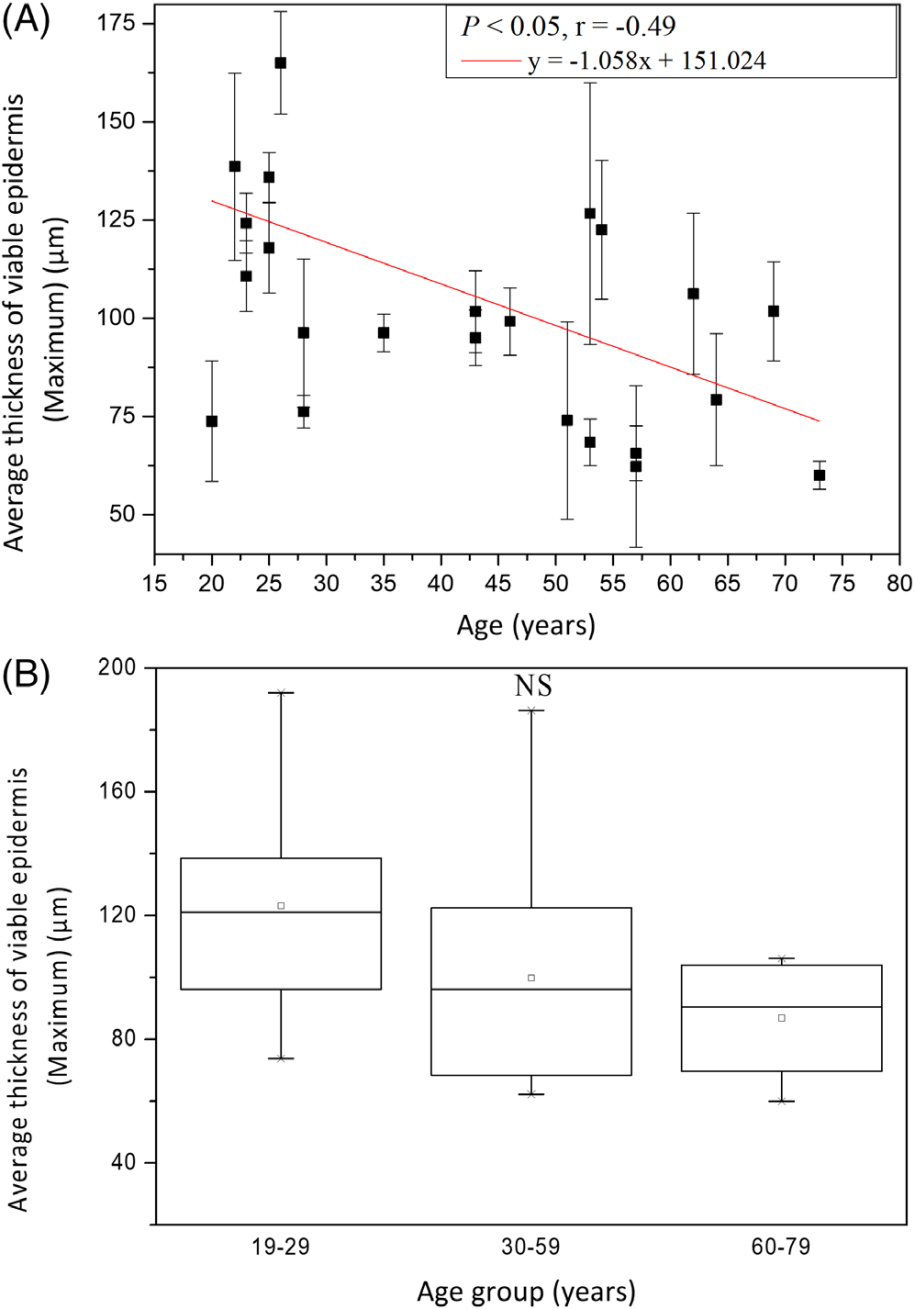
The maximum thickness of viable epidermis vs age from the ventral forearms of Caucasian subjects (n = 25). A, In simple linear regression analysis, *P* = .01. Correlation coefficient *r* for age in Caucasians = −.49. B, In Kruskal‐Wallis test, *P* = .14 among the three age groups. NS, not significant

The maximum thickness of viable epidermis (*T*_VEM_) for age groups 19‐29, 30‐59 and 60‐79 years were 123.05 ± 37.18 μm, 99.8 ± 36.03 μm and 86.8 ± 21.41 μm, respectively, without statistically significant difference in Kruskal‐Wallis test for Caucasian subjects (*P* = .14). For the maximum thickness of viable epidermis, simple linear regression however showed statistically significant correlation with age in Caucasian subject (Figure 5; *P* = .01). Therefore, the maximum thickness of viable epidermis slightly decreased with age.

The result in Kruskal‐Wallis test implied that the minimum thickness of viable epidermis remained the same with age.

The maximum thicknesses of viable epidermis seemed to decrease with age, but differences between age groups were not significant. Both results of the minimum and the maximum thickness implied that not the thickness of viable epidermis but the depth of rete ridge might decrease with age. This implication will be confirmed later in the analysis of the depth of dermal papilla zone, which is the same as the depth or length of rete ridge.

#### 3.1.2 Average area of granular cells

The average area of the granular cells (A_G_) was 541.7 ± 166.76 μm^2^ at 19 to 29 years, 674.66 ± 196.61 μm^2^ at 30 to 59 years, and 648.98 ± 218.75 μm^2^ at 60 to 79 years, respectively, without statistically significant difference in anova for Caucasian subjects (*P* = .21). For cellular size, simple linear regression showed no statistically significant correlation with age in Caucasian subject (Table 3; *P* = .06).

**Figure 6:**
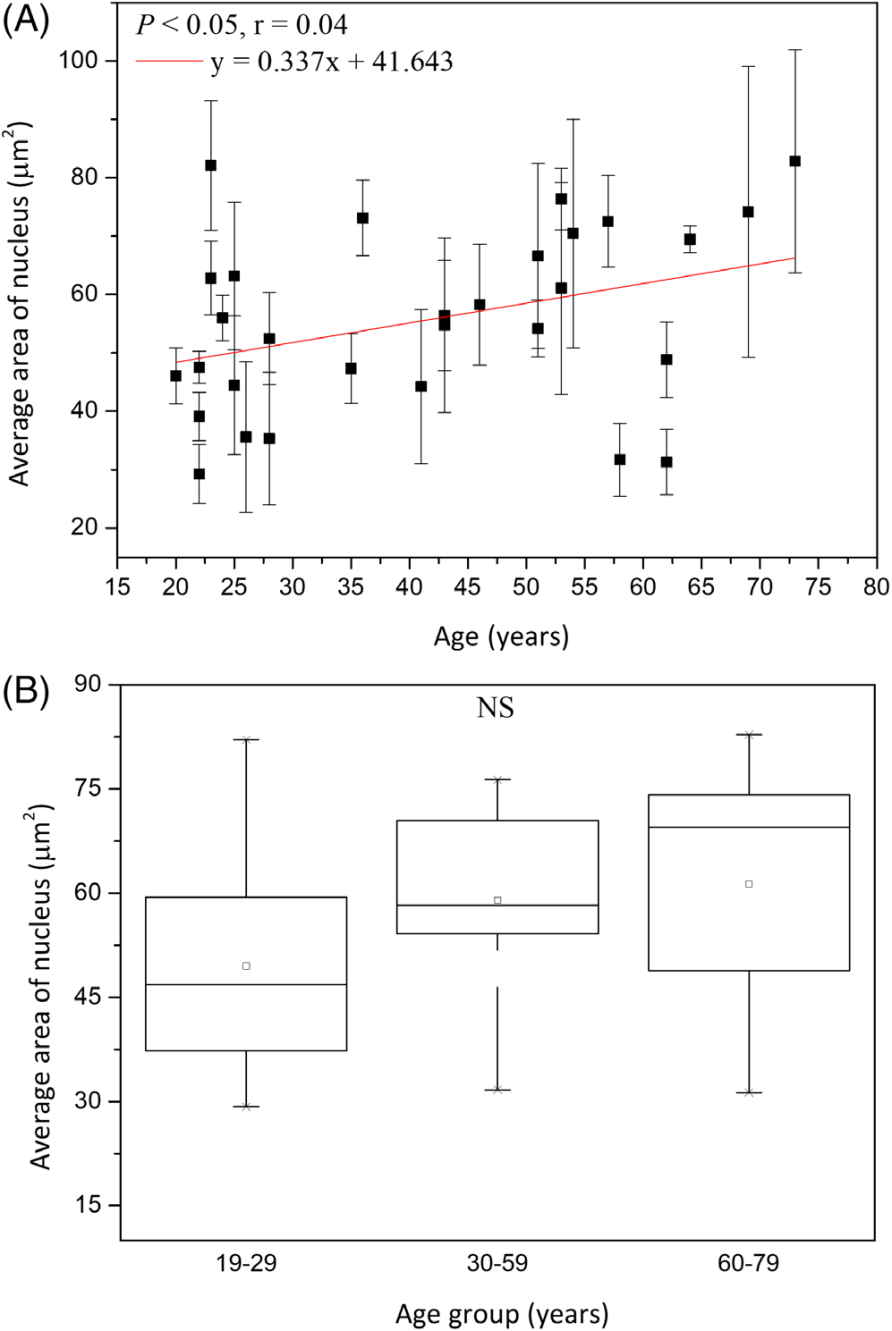
Average area of granular nucleus vs age from the ventral forearms of Caucasian subjects (n = 30). A, In simple linear regression, *P* < .05. B, In ANOVA, *P* = .21 among the three age groups. NS, not significant

The average area of the granular nucleus (*A*_GN_) was 49.49 ± 14.87 μm^2^ at 19 to 29 years, 58.99 ± 12.99 μm^2^ at 30 to 59 years, and 61.30 ± 20.92 μm^2^ at 60 to 79 years, respectively, without statistically significant difference in anova for Caucasian subjects (*P* = .21). For nuclear size, simple linear regression however showed statistically significant correlation with age in Caucasian subject (Figure 6; *P* < .05). In combination, the average area of the granular nucleus slightly increased with age, but differences between age groups were not significant. In Figure 6, tendency of increasing with age could be observed, but data were somehow scattered in Caucasians.

The NC ratio of granular cells (NC_G_) was 0.10 ± 0.01 at 19 to 29 years, 0.1 ± 0.02 at 30 to 59 years and 0.11 ± 0.02 at 60 to 79 years, respectively, without statistically significant difference in anova for Caucasian subjects (*P* = .85). For NC ratio, simple linear regression showed no statistically significant correlation with age in Caucasian subject (Table 3; *P* = .74). The average NC ratio of granular cells from all the subjects was 0.10 ± 0.02.

#### 3.1.3 Average area of basal cells

**Figure 7:**
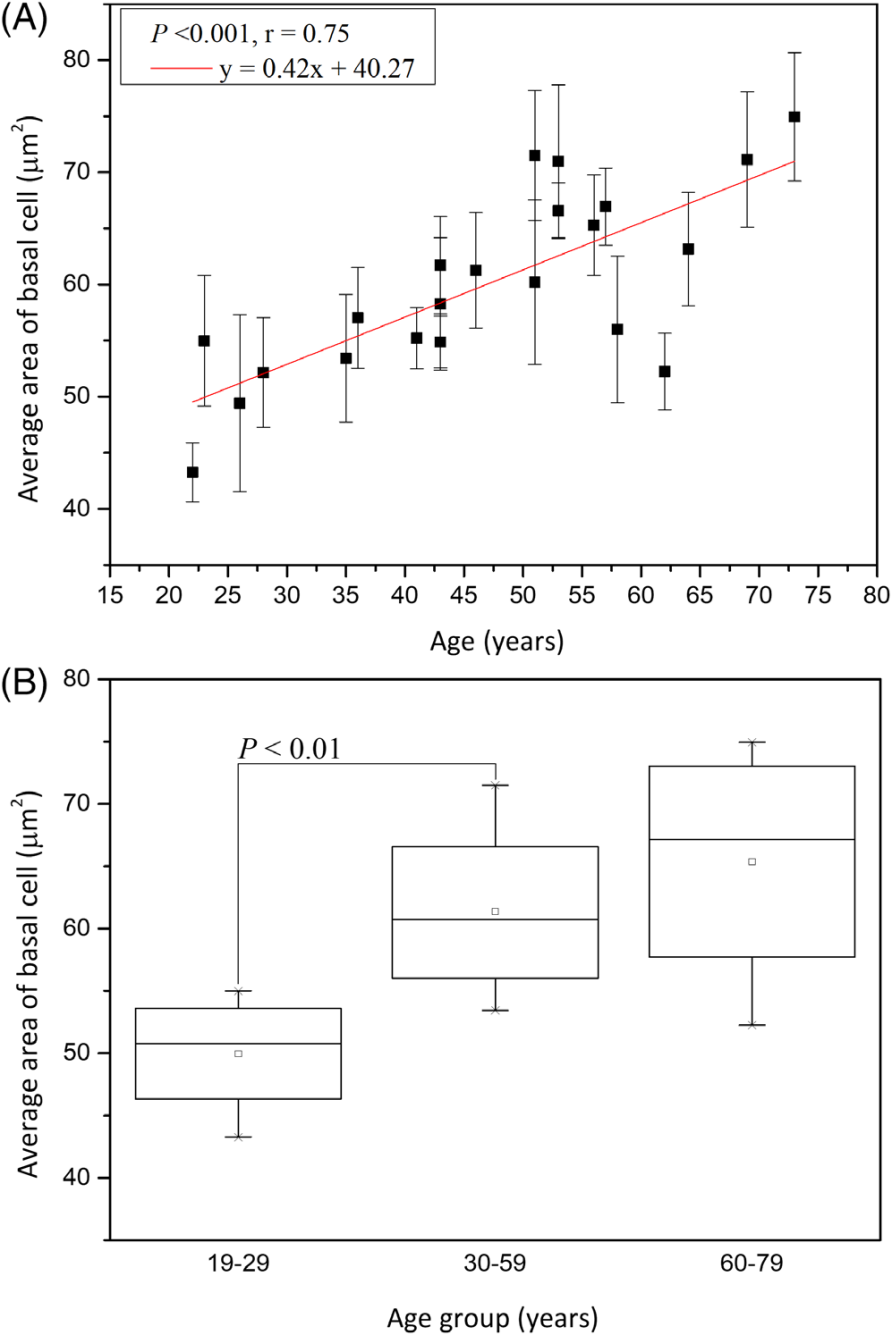
Cellular size of the basal cells vs age from the ventral forearms of Caucasian subjects (n = 22). A, In simple linear regression, *P* < .001. Correlation coefficient *r* = 0.75. B, In Kruskal‐Wallis test, *P* = .02 among the three age groups. NS, not significant

The average area of the basal cells (A_B_) was 49.96 ± 5.01 μm^2^ at 19 to 29 years, 61.38 ± 6.01 μm^2^ at 30 to 59 years, and 65.37 ± 10.03 μm^2^ at 60 to 79 years, respectively, with statistically significant difference in Kruskal‐Wallis test for Caucasian subjects (Figure 7; *P* = .02). For average area of the basal cells, simple linear regression showed statistically significant correlation with age in Caucasian subject (Figure 7; *P* < .001). The cellular size was positively related to age with a correlation coefficient *r* = .75.

**Figure 8:**
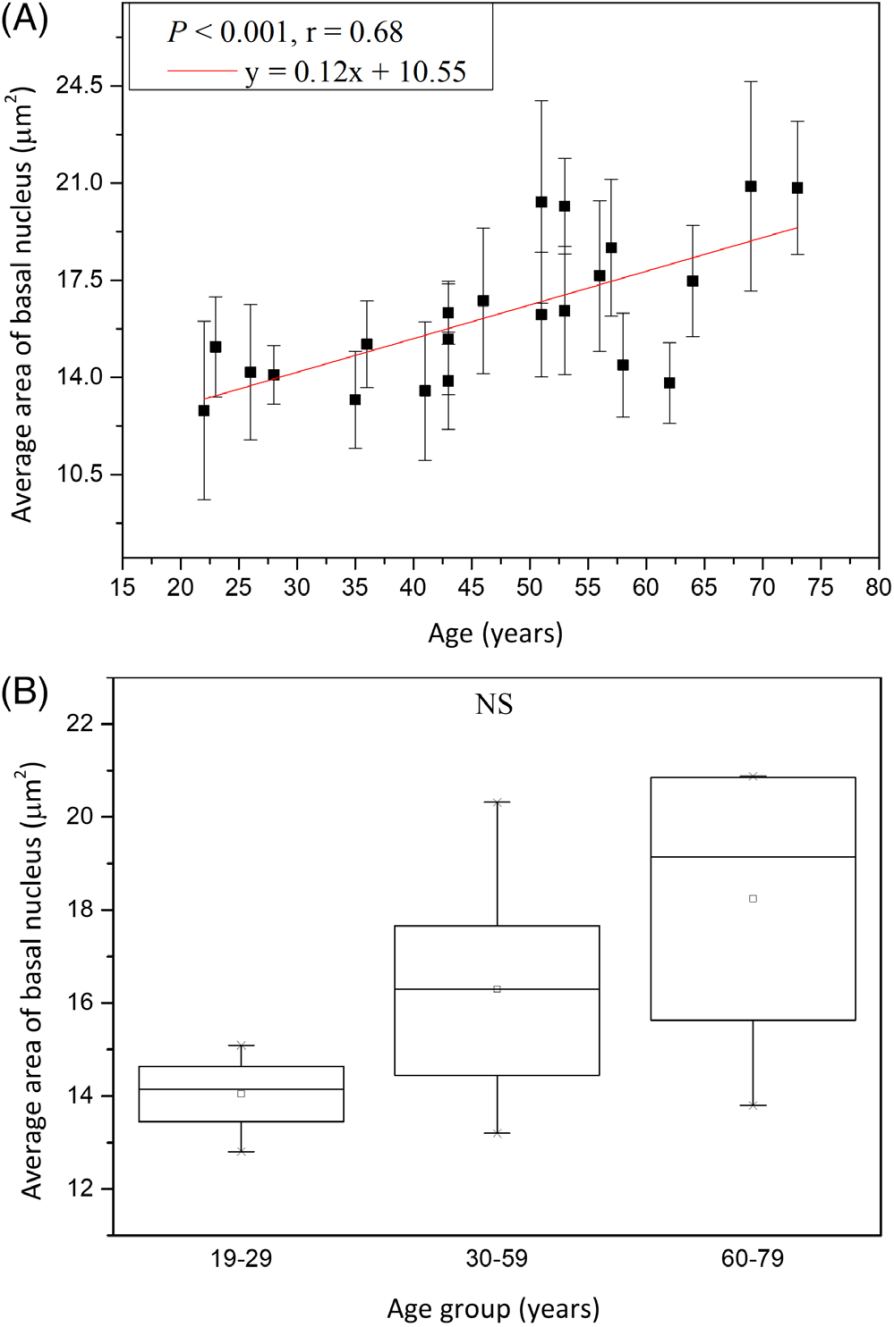
Nucleus size of the basal cells vs age from the ventral forearms of Caucasian subjects (n = 22). A, In simple linear regression, *P* < .001. Correlation coefficient *r* = .68. B, In Kruskal‐Wallis test, *P* = .09 among the three age groups. NS, not significant

The average area of the basal nucleus (*A*_BN_) was 14.04 ± 0.94 μm^2^ at 19 to 29 years, 16.30 ± 2.28 μm^2^ at 30 to 59 years, and 18.24 ± 3.37μm^2^at 60 to 79 years, respectively, without statistically significant difference in Kruskal‐Wallis test for Caucasian subjects (Figure 8; *P* = .09). For average area of the basal nucleus, simple linear regression however showed statistically significant correlation with age in Caucasian subject (Figure 8; *P* < .001). The nucleus size was positively related to age with *r* = .68.

The NC ratio of the basal cells (NC_B_) remained consistent among different‐aged groups in Kruskal‐Wallis test for Caucasian subjects (*P* = .55). The NC ratio of basal cell was 0.35 ± 0.09 at the age of 19 to 29 years, 0.36 ± 0.02 at 30 to 59 years, and 0.35 ± 0.01 at 60 to 79 years. Moreover, for the NC ratio, simple linear regression showed no statistically significant correlation with age in Caucasian subject (Table 3; *P* = .55). The average NC ratio of basal cells from all the subjects was 0.35 ± 0.013.

In summary, the cellular and nuclear area of basal cells, but not the NC ratio of basal cells, was increased with advancing age.

### 3.2 In vivo analysis of dermal papillae within dermal papilla zone for Caucasians

#### 3.2.1 Depth of dermal papilla zone

**Figure 9:**
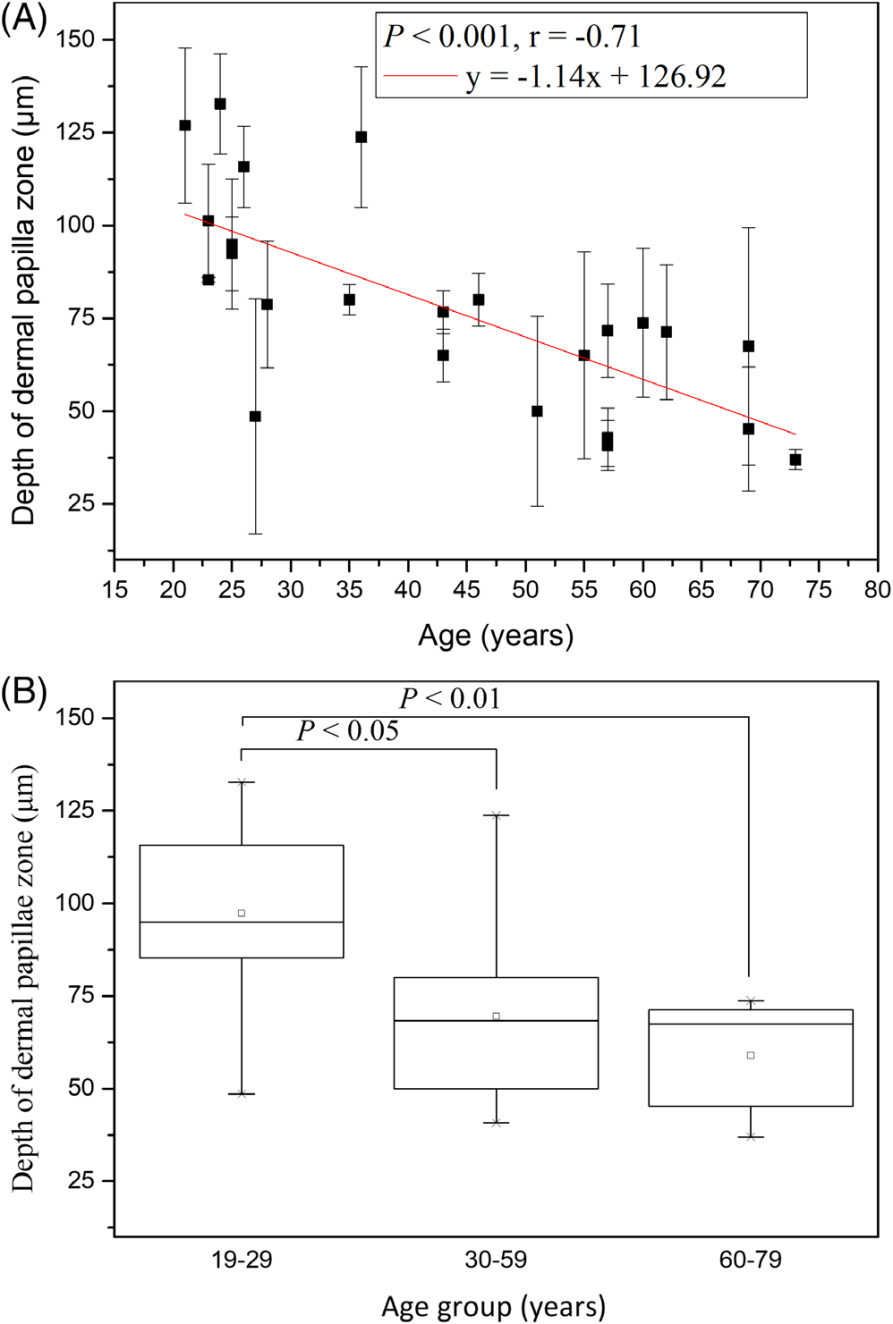
Depth of dermal papillae zone vs age from the ventral forearms of Caucasian subjects (n = 24). A, In linear regression analysis, *P* < .001. Correlation coefficient *r* = −.71. B, In Kruskal‐Wallis test, *P* = .01 among the three age groups

In Kruskal‐Wallis test of the depth of dermal papilla zone (*T*_DPZ_), the depths for age groups 19‐29, 30‐59 and 60‐79 years were 97.41 ± 25.93 μm, 69.59 ± 23.96 μm, and 58.97 ± 16.70 μm, respectively, and the effect of age group in Caucasian subjects was statistically significant (Figure 9; *P* = .01; Kruskal‐Wallis test). Moreover, the Mann‐Whitney U test showed statically significance between the 19‐29 group and the 30‐59 group, and also showed significance between the 19‐29 group and the 60‐79 group. The simple linear regression analysis was also statistically significant for age in Caucasian subjects (Figure 9; *P* < .001). The correlation was negative (*r* = −.71) for *T*_DPZ_ to age. The result of thinner dermal papilla zone in the aged Caucasian skin indicated that the dermal‐epidermal junction flattened with aging.

#### 3.2.2 Dermal papillae volume per unit area

**Figure 10:**
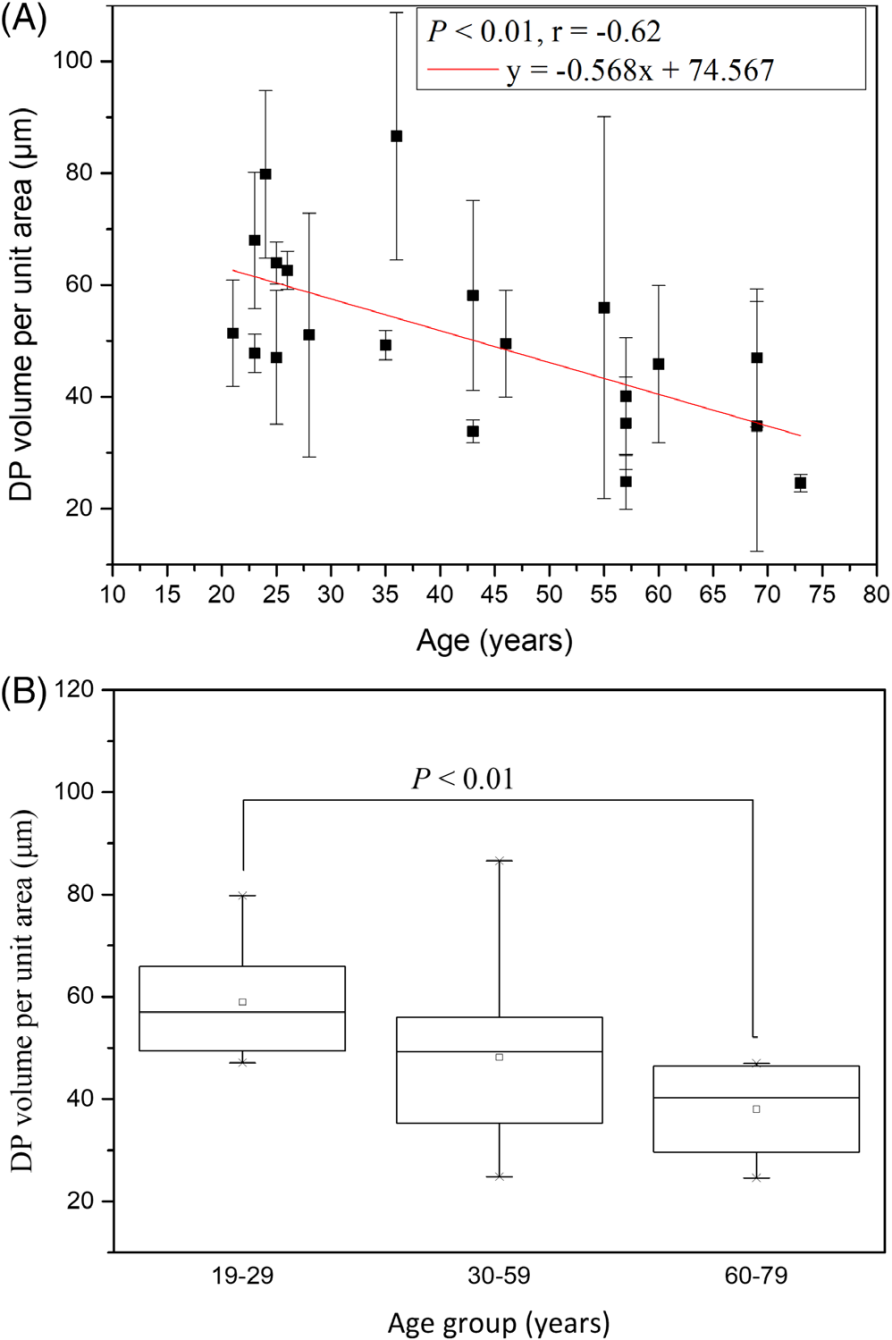
DP volume per unit area vs age from the ventral forearms of Caucasian subjects (n = 21). A, In simple linear regression analysis, *P* = .002. Correlation coefficient *r* = −.62. B, In Kruskal‐Wallis test, *P* = .03 among the three age groups

The dermal papillae volume per unit area (V) for age groups 19‐29, 30‐59, and 60‐79 years were 58.96 ± 11.58 μm, 48.16 ± 18.10 μm, and 38.05 ± 10.54 μm, respectively, and the effect of age group in Caucasian subjects was statistically significant (Figure 10; *P* = .03, Kruskal‐Wallis test). The simple regression analysis was also statistically significant for age in Caucasians (Figure 10; *P* = .002). The correlation was negative (*r* = −.62) for the dermal papillae volume per unit area to age in Caucasians. In Figure 10, the tendency of decrease with age for Caucasian subject could be observed.

#### 3.2.3 Dermal papillae volume ratio within dermal papilla zone

**Figure 11:**
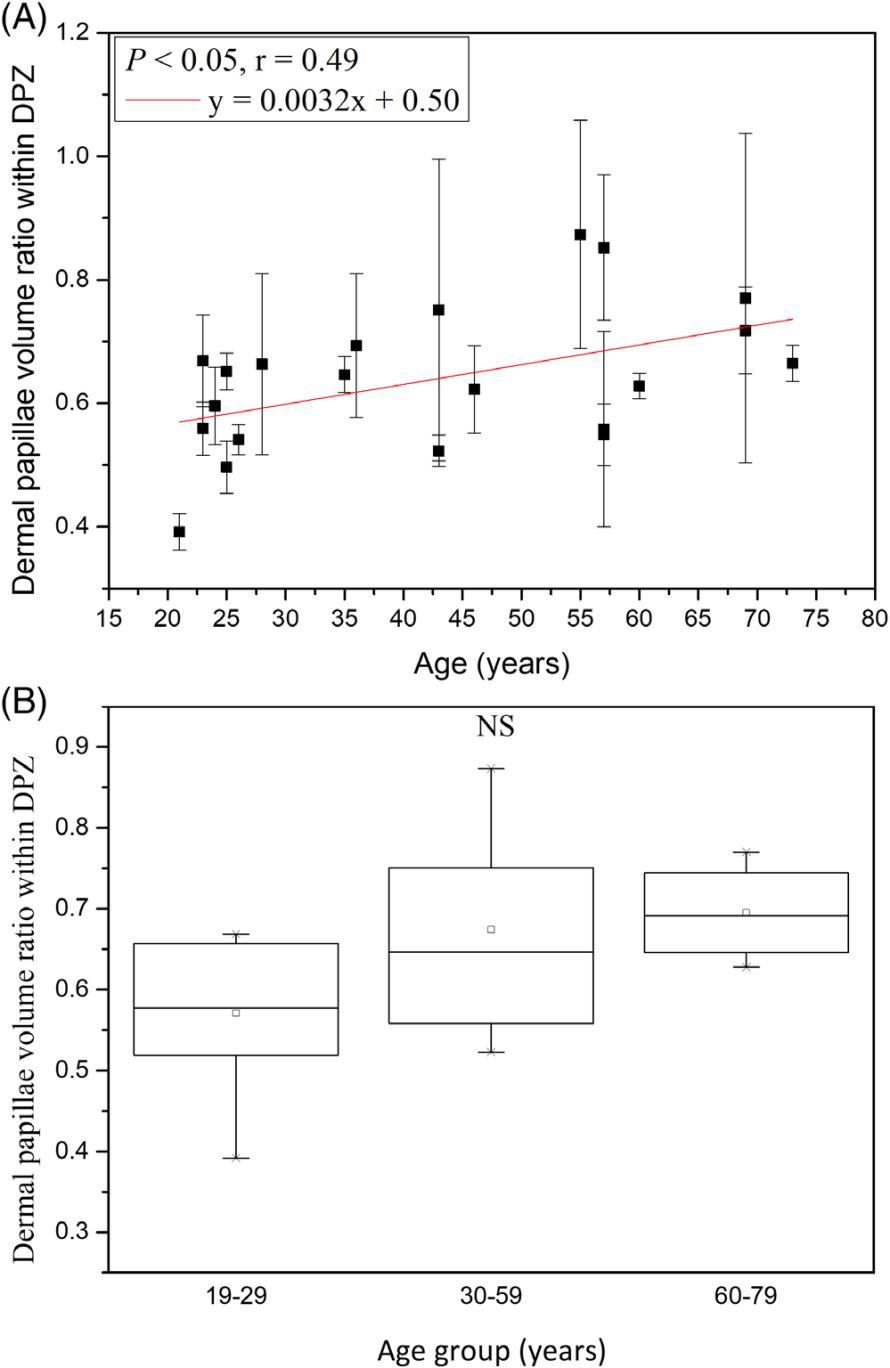
Dermal papillae volume ratio within dermal papilla zone vs age from the ventral forearms of Caucasian subjects (n = 21). A, In simple linear regression analysis, *P* = .03. Correlation coefficient *r* = 0.49. B, In Kruskal‐Wallis test, *P* = .13 among the three age groups. NS, not significant

The dermal papilla volume ratios within DPZ (R) for age groups 19 to 29, 30 to 59 and 60 to 79 years were 59.65 ± 6.73%, 67.43 ± 12.9% and 69.51 ± 6.23%, respectively, with no statistically significant difference in Kruskal‐Wallis test for Caucasian subjects (Figure 11; *P* = .13). However, we on the other hand found that the dermal papillae volume ratio within DPZ was positively correlated to age with significance in the simple linear regression analysis (Figure 11; *P* = .03; *r* = .49). It meant that dermal papillae occupied more proportion of volume in the dermal papilla zone as age increased.

It could be noticed that the dermal papillae volume ratio within dermal papilla zone (*V*_total_/[OA_max_ × *T*_DPZ_]) was the dermal papillae volume per unit area (*V*_total_/OA_max_) divided by the depth of dermal papilla zone (*T*_DPZ_). For Caucasian subjects, it was found that the dermal papillae volume per unit area was slowly decreased with increasing age and the depth of dermal papilla zone rapidly decreased with increasing age, so the increase with age of the dermal papillae volume ratio within dermal papilla zone was quite reasonable.

The result in Kruskal‐Wallis test implied that the dermal papillae volume ratio within dermal papilla zone among age groups changed without statistical significance.

#### 3.2.4 3D interdigitation index

**Figure 12:**
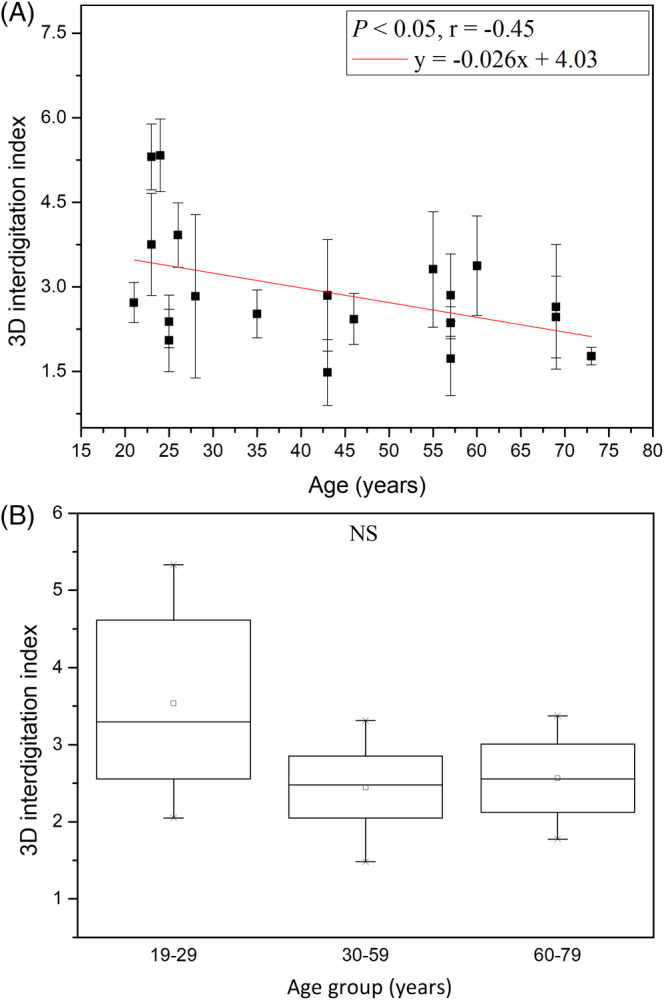
3D interdigitation index vs age from the ventral forearms of Caucasian subjects (n = 20). A, In simple linear regression analysis, *P* = .04. Correlation coefficient r = −.45. B, In Kruskal‐Wallis test, *P* = .20 among the three age groups. NS, not significant

The 3D interdigitation index (I) for age groups 19 to 29, 30 to 59 and 60 to 79 years were 3.65 ± 1.32, 2.44 ± 0.60 and 2.56 ± 0.66, respectively, with no statistically significant difference in Kruskal‐Wallis test for Caucasian subjects (Figure 12; *P* = .20). However, simple linear regression analysis showed that the correlation between the 3D interdigitation index and age was negative with statistical significance in Caucasian subjects (Figure 12; *P* = .04; *r* = −.45). For Caucasian subjects, the interface area of dermal papillae became smaller with advancing age, which revealed the undulation of dermal‐epidermal junction was smaller in the elder skin.

The result in Kruskal‐Wallis test implied that the 3D interdigitation index decreases with advancing age.

#### 3.2.5 2D collagen density in DP

The 2D collagen density in DP(D) for age groups 19 to 29, 30 to 59 and 60 to 79 years were 0.33 ± 0.05, 0.30 ± 0.07, and 0.34 ± 0.05, respectively, without statistically significant difference in Kruskal‐Wallis test for Caucasian subjects (*P* = .58). Moreover, simple linear regression analysis showed no statistically significant correlation between the 2D collagen density and age for Caucasian subjects (Table 3; *P* = .83). Our experiment showed that the 2D collagen density in DPZ did not change with age.

Both results in Kruskal‐Wallis test and anova implied that the 2D collagen density in DP did not change with age.

#### 3.2.6 Collagen volume in DP per unit area

**Figure 13:**
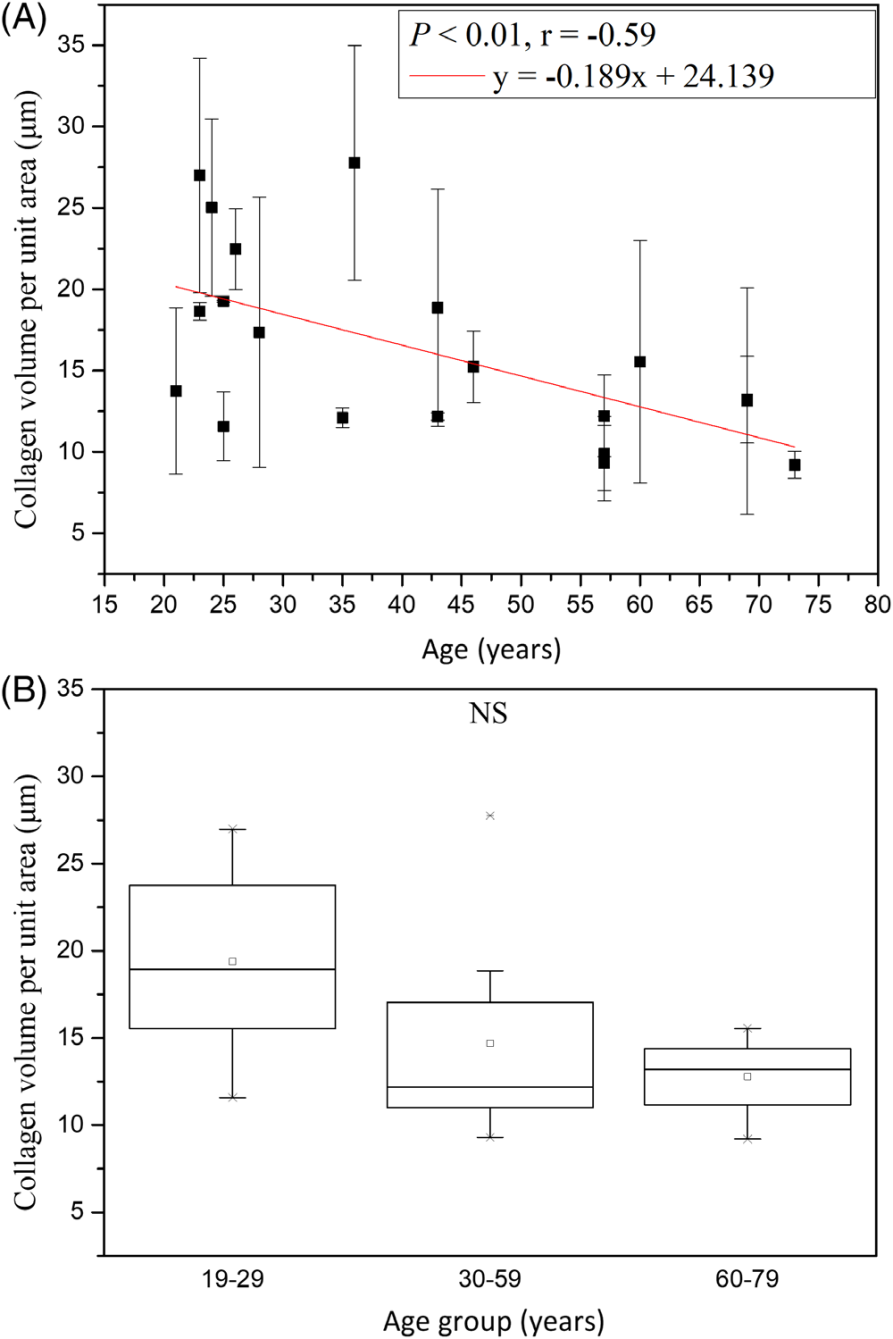
Collagen volume per unit area vs age from the ventral forearms of Caucasian subjects (n = 20). A, In simple linear regression analysis, *P* = .006. Correlation coefficient *r* = −.59. B, In Kruskal‐Wallis test, *P* = .11 among the three age groups. NS, not significant

The collagen volume in DP per unit area for age groups 19 to 29, 30 to 59 and 60 to 79 years were 20.18 ± 5.17, 14.70 ± 6.09 and 12.78 ± 2.63, respectively, without statistically significant difference in Kruskal‐Wallis test for Caucasian subjects (Figure 13; *P* = .11). However, simple linear regression analysis showed a statistically significant correlation between the collagen volume per unit area and age for Caucasian subjects (Figure 13; *P* = .006; *r* = −.59). Our experiment showed that the collagen volume per unit area in DPZ decreased with age.

### 3.3 Comparative analysis of viable epidermis between Caucasian and Asian

#### 3.3.1 Viable epidermis thickness

Simple linear regression analysis showed no significant correlation between the minimum thickness of viable epidermis and age for Caucasian and Asian subjects 13. anova showed significant difference in the minimum thickness of viable epidermis for Asian subjects, but on the other hand, Kruskal‐Wallis test showed no significant difference in the minimum thickness of viable epidermis for Caucasian subjects.

Analysis of variance showed no significant difference in the minimum thickness of viable epidermis between Caucasian and Asian subjects (*P* for race = .13), and the interaction between age and race showed no significances (Table 3, *P* for age*race = .73).

**Figure 14:**
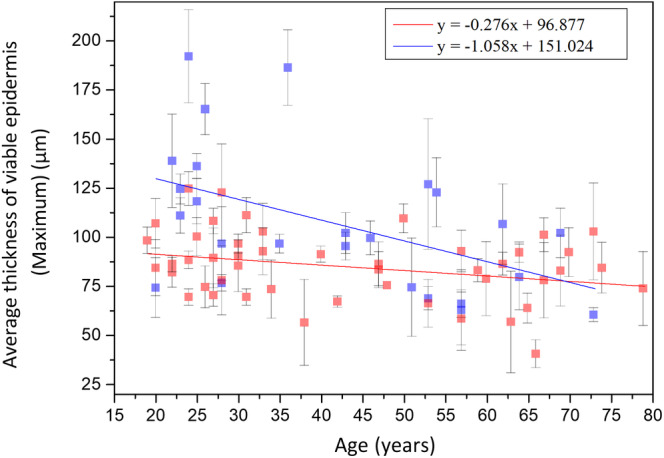
Maximum thickness of viable epidermis vs age from the ventral forearms of Caucasian and Asian subjects. In ancova, *P* value for age*race = .02. Data of Caucasian (blue) and Asian (red) subjects were separately displayed

Simple linear regression analysis showed negative correlations between the maximum thickness of viable epidermis and age for Caucasian and Asian subjects 13. anova showed no significant difference in the maximum thickness of viable epidermis for Asian subjects 13. Moreover, ancova showed significant difference in the interaction between age and race (Figure 14; *P* for age*race = .02).

The results in ancova implied that the minimum thickness remained with age with similar trends between Caucasian and Asian subjects while the maximum thickness for Caucasian subjects decreased with age with a faster rate compared with that for Asian subjects.

#### 3.3.2 Average area of granular cells

**Figure 15:**
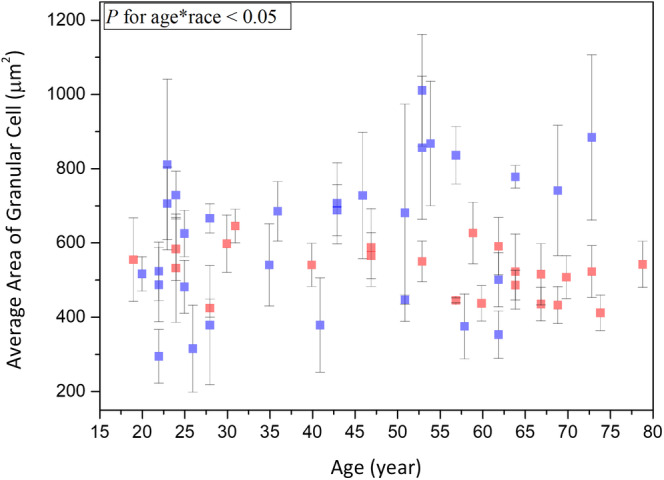
Average area of granular cells vs age from the ventral forearms of Caucasian and Asian subjects. In ancova, *P* value for race = .01; *P* value for age*race = .02. Data of Caucasian (blue) and Asian (red) subjects were separately displayed

Simple linear regression analysis showed no significant correlation between the cellular size of granular cells and age for Caucasian and Asian subjects 13. Moreover, ancova showed a statistically significant difference in the cellular size between Caucasian and Asian subjects (Figure 15; *P* for race = .01), and the interaction between age and race showed significances (Figure 15; *P* for age*race = .02). The reason for the huge interaction between age and race is that a trend of increase with age could be observed in the cellular size of Caucasian subjects while the cellular size of Asian subjects did not change with age, as shown in Figure 15.

**Figure 16:**
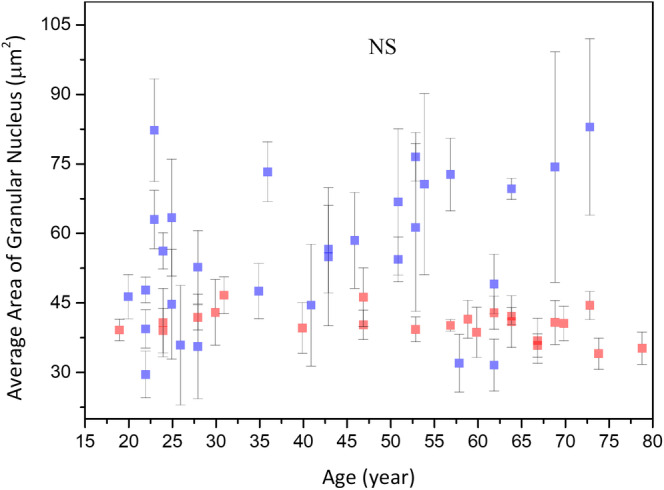
Average area of granular nucleus vs age from the ventral forearms of Caucasian and Asian subjects. In ancova, *P* value for race = .005; *P* value for age*race = .03. Data of Caucasian (blue) and Asian (red) subjects were separately displayed. NS, not significant

Simple linear regression analysis showed positive correlation between the nuclear size of granular cells and age for Caucasian subjects. But between the nuclear size of granular cells and age for Asian subjects, no significant correlation could be observed. anova showed no significant difference in the nuclear size of granular cells for Caucasian and Asian subjects. In combination with our previous results on Asian skin, ancova showed a statistically significant difference in the nuclear size between Caucasian and Asian subjects (Figure 16; *P* for race = .005), and the interaction between age and race also showed significances (Figure 16; *P* for age*race = .03). The reason why the interaction between age and race is huge is that a positive correlation between the nuclear size of Caucasian subjects and age could be observed while the nuclear size of Asian subjects did not change with age, as shown in Figure 16.

In this study, we recalculated the NC ratio of granular cells for Asian subjects. The recalculated NC ratio of granular cells for Asian subjects was 0.0891 ± 0.0202 at 19 to 29 years, 0.0822 ± 0.00958 at 30 to 59 years and 0.0892 ± 0.0138 at 60 to 79 years, respectively, without statistically significant difference in anova for Asian subjects (*P* for age group in Asians = .41). For NC ratio, simple linear regression showed no statistically significant correlation with age in Asian subject (*P* for age in Asians = .63). On the other hand, simple linear regression analysis also showed no significant correlation between the NC ratio of granular cells and age for Caucasian subjects. The NC ratio of granular cells for Caucasian subjects among three age groups showed no difference in anova. In combination with our recalculated results on Asian skin, ancova showed a statistically significant difference in the NC ratio between Caucasian and Asian subjects (*P* for race = .02), but the interaction between age and race showed no significances (Table 3; *P* for age*race = .44).

#### 3.3.3 Average area of basal cells

**Figure 17:**
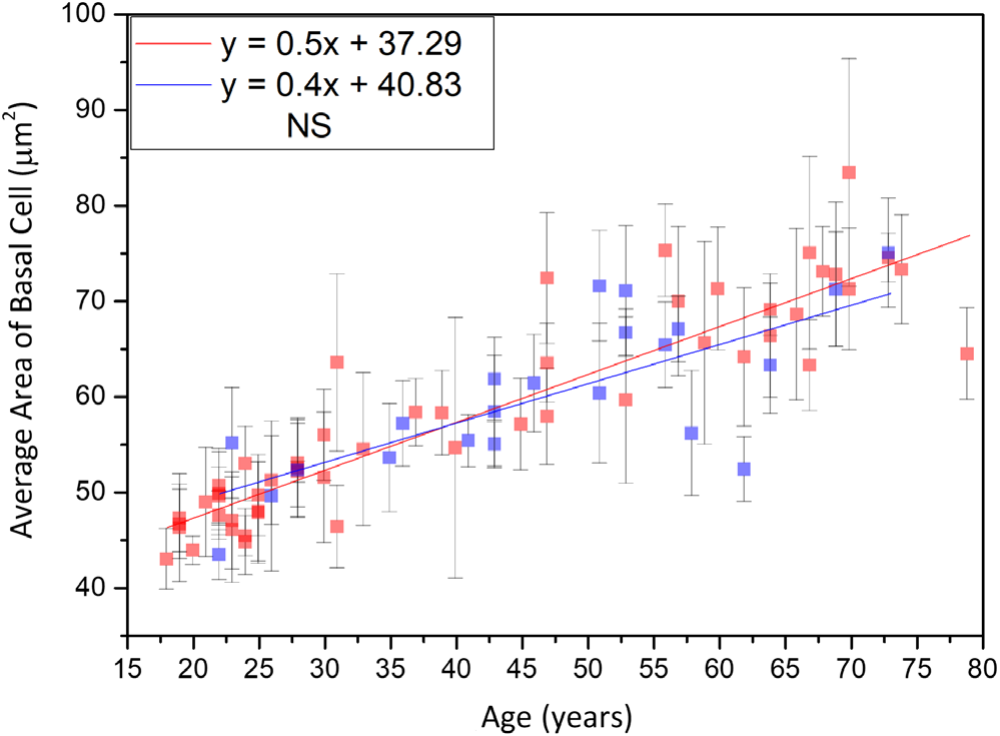
Cellular size of the basal cells vs age from the ventral forearms of Caucasian Asian subjects. In ancova, *P* value for race = .61; *P* value for age*race = .32. Data of Caucasian (blue) and Asian (red) subjects were separately displayed. NS, not significant

Simple linear regression analysis showed positive correlation between the cellular size of basal cells and age for Caucasian and Asian subjects 13. Moreover, for the cellular size between Caucasian and Asian, ancova showed no significant difference (Figure 17; *P* for race = .61).

**Figure 18:**
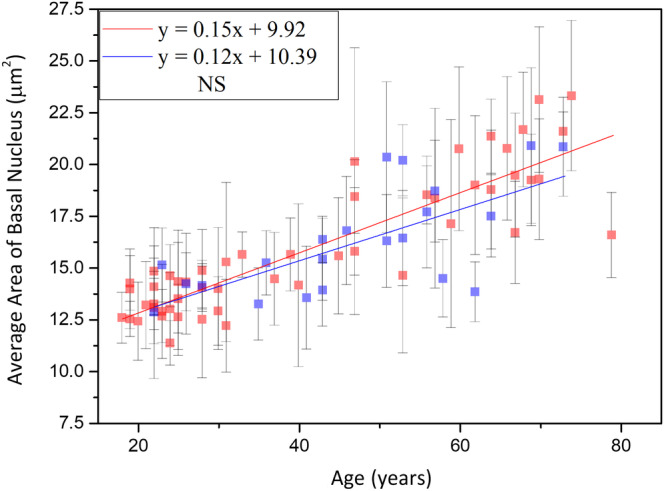
Nucleus size of the basal cells vs age from the ventral forearms of Caucasian and Asian subjects. In ancova, *P* value for race = .24; *P* value for age*race = .39. Data of Caucasian (blue) and Asian (red) subjects were separately displayed. NS, not significant

Simple linear regression analysis showed positive correlation between the nuclear size of basal cells and age for Caucasian and Asian subjects 13. Moreover, for the nuclear size between Caucasian and Asian, ancova showed no significant difference (Figure 18; *P* for race = .24).

There were no statistical significances of the interaction between age and race for the cellular and nuclear size of the basal cells in ancova (for the interaction between age and race, *P*_cell_ = 0.32, and *P*_nucleus_ = .39).

Simple linear regression analysis showed no significance between the NC ratio of basal cells and age for Caucasian and Asian subjects. Moreover, ancova showed no statistically significant difference in the NC ratio between Caucasian and Asian subjects, and the interaction between age and race showed no significances (*P* for age*race = .58).

### 3.4 Comparative analysis of dermal papillae within dermal papilla zone between Caucasian and Asian

#### 3.4.1 Depth of dermal papilla zone

**Figure 19:**
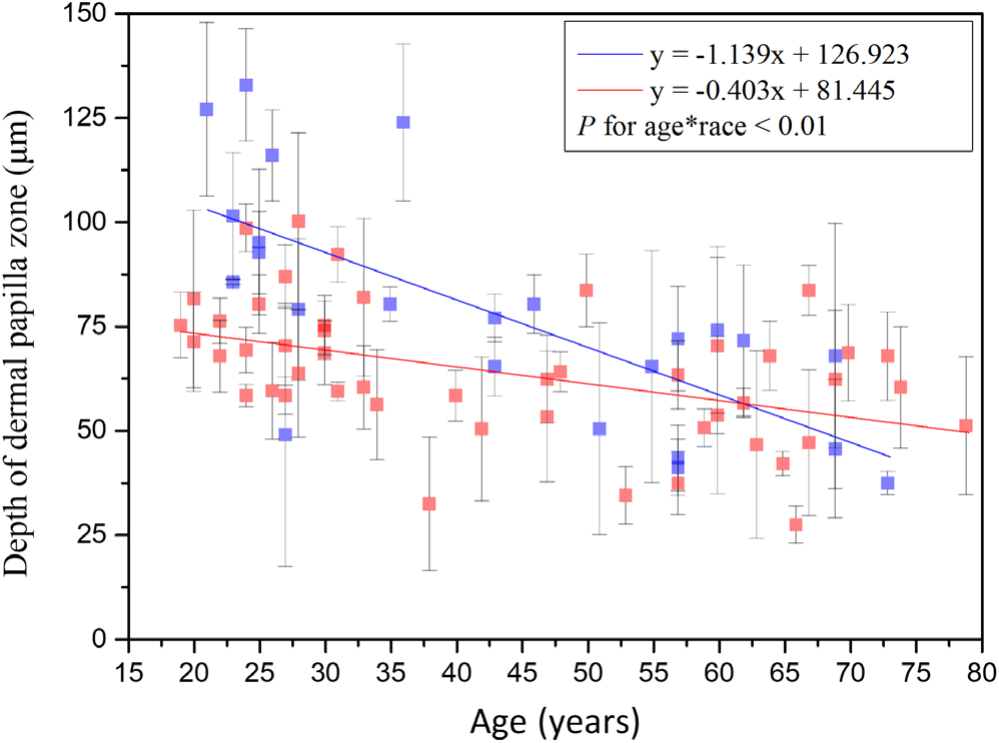
Depth of dermal papillae zone vs age from the ventral forearms of Caucasian and Asian subjects. *P* value for age*race = .003. Data of Caucasian (blue) and Asian (red) subjects were separately displayed

Simple linear regression analysis showed negative correlation between the depth of dermal papilla zone and age for Caucasian and Asian subjects 14. In addition, ANCOVA showed statically significant difference in the interaction between age and race (Figure 19; *P* for age*race = .003). That is, the depth of dermal papilla zone for Caucasian subjects decreased at a much faster rate compared with that for the Asian subjects.

#### 3.4.2 Dermal papillae volume per unit area

**Figure 20:**
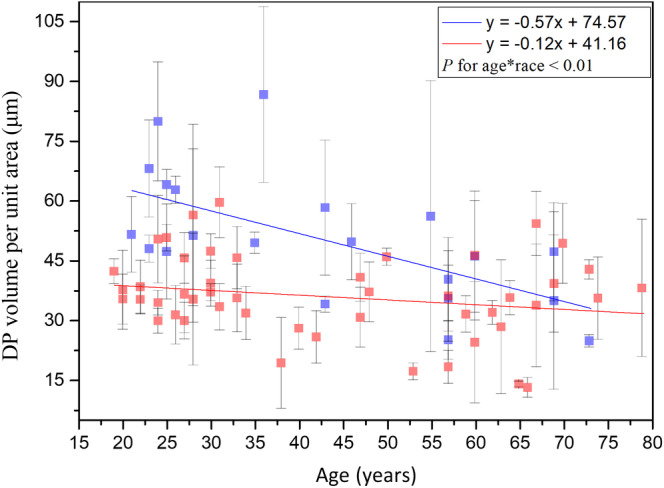
DP volume per unit area vs age from the ventral forearms of Caucasian and Asian subjects. In ancova, *P* value for age*race = .009. Data of Caucasian (blue) and Asian (red) subjects were separately displayed

Simple linear regression analysis showed negative correlation between the dermal papillae volume per unit area and age for Caucasian subjects, but it showed no significance between that for Asian subjects 14. In addition, the interaction between age and race showed significance in ancova (Figure 20; *P* for age*race = .009). That is, the dermal papillae volume per unit area for Caucasian subjects decreased at a much faster rate compared with that for the Asian subjects.

#### 3.4.3 Dermal papillae volume ratio within dermal papilla zone

Simple linear regression analysis showed positive correlation between the dermal papillae volume ratio within DPZ and age for Caucasian and Asian subjects. In combination with our previous results on Asian skin, ancova showed a statistically significant difference in the dermal papillae volume ratio within DPZ between Caucasian and Asian subjects (*P* for race <.001), but the interaction between age and race showed no significances (Table 3; *P* for age*race = .17).

#### 3.4.4 3D interdigitation index

Simple linear regression analysis showed negative correlation between the 3D interdigitation index and age for Caucasian and Asian subjects 14. However, ancova showed no statistically significant difference in the interaction between age and race (Table 3; *P* for age*race = .199). Comparing between Caucasian and Asian subjects, there was no statistically significant difference of aging trend in the 3D interdigitation index.

#### 3.4.5 2D collagen density in DP

In 2D collagen density in DP analysis, we used an algorithm of newer version compared with the version we used on Asian subjects 14, so we recalculated the 2D collagen density for Asian subjects. For 2D collagen density in DP, simple linear regression showed no statistically significant correlation with age in Asian subject (*P* for age in Asians = .18). On the other hand, simple linear regression analysis also showed no significant correlation between the 2D collagen density and age for Caucasian subjects. In combination with our recalculated results on Asian skin, ancova showed no significant difference in the 2D collagen density in DP between Caucasian and Asian subjects, and the interaction between age and race showed no significance (Table 3; *P* for race = .35 and *P* for age*race = .41). Our experiment showed that the 2D collagen density in DP does not change with race.

#### 3.4.6 Collagen volume in DP per unit area

We obtained the collagen volume in DP per unit area for Asian subjects by multiplying the 2D collagen density by DP volume per unit area for Asian subjects 14. For collagen volume in DP per unit area, simple linear regression showed negative correlation with age in Asian subject (*P* for age in Asians = .0855). On the other hand, simple linear regression analysis showed also negative correlation between the collagen volume in DP per unit area and age for Caucasian subjects. ancova showed a statistically significant difference in the collagen volume between Caucasian and Asian subjects, but the interaction between age and race showed no significance (Table 3, *P* for race = .0005 and *P* for age*race = .12). Our experiment showed that the collagen volume in DP per unit area varies with race, but not the 2D collagen density in DP.

## 4 DISCUSSION AND CONCLUSION

In this study, some morphological changes in the skin of Asian subjects have already been acquired previously and their relationships with intrinsic skin aging have been published 13, 14. As for Caucasian skin aging studies and the comparison between the aging in different races, here we review previous studies for Caucasian subjects and compare our results in this study with our previous study for Asian subjects.

### 4.1 Comparison between results of viable epidermis thickness

Several previous studies have repeatedly investigated the epidermis thickness in several body sites in Caucasian subjects by biopsy and in vivo techniques. However, the thickness of the viable epidermis was measured in only a few studies or was studied indirectly, that is, by measuring both the stratum corneum and the entire epidermis to determine the age‐related changes in the viable epidermis.

In the study of Koehler et al 30, the viable epidermis thickness of the dorsal forearm of 30 German volunteers was not found to be different between age groups based on in vivo multiphoton laser tomography.

Our conclusion was on the other hand consistent with the conclusion made by Neerken et al 31, who separately measured the minimum and maximum epidermis thicknesses of the volar forearm of 30 Caucasian volunteers using confocal laser scanning microscopy. They concluded that the overall effect of aging skin was found to be a significant decrease in the maximum epidermis thicknesses, whereas the minimum epidermis thicknesses changed only slightly with age.

The study results of the viable epidermis thickness imply that the maximum epidermis thickness in the Caucasian subjects decreases with age with a faster trend when compared with that in the Asian subjects and the Caucasian subjects have on average a thicker viable epidermis compared to that in Asian subjects. Our study also implies that not only the maximum thickness of viable epidermis but also the depth of rete ridge decreases with age. The difference between the average maximum thickness of viable epidermis of two extreme groups was 36.3 μm, which is close to the difference between the average DPZ depth of two extreme groups, 38.4 μm. In our study, including the previous measurements of the viable epidermis thickness and DPZ depth in Asian subjects 13, 14, we thus infer that not the viable epidermis itself but the depth of rete ridges decreased in the aged skin.

### 4.2 Comparison between results of average area of granular cells

In this study, no statistically significant differences were observed in the cellular size, the nuclear size and the NC ratio of granular cells among the three different age groups of Caucasian subjects. These results are consistent with those of a recent study conducted based on in vivo multiphoton laser tomography, where Koehler et al found that the cellular size and the nuclear size of dorsal forearm remained constant in the stratum granulosum among different age groups 30. In their study, the average areas of the granular cells for mean ages 23.4, 47.3 and 72.1 years were 299 ± 52 μm^2^, 295 ± 48 μm^2^ and 321 ± 65 μm^2^; the average areas of the granular nucleus for mean ages 23.4, 47.3, and 72.1 years were 37.1 ± 3 μm^2^, 34.4 ± 5.4 μm^2^ and 39.0 ± 8.1μm^2^. By using the data provided above, the NC ratio of granular cells for mean ages 23.4, 47.3 and 72.1 years were 0.142, 0.132 and 0.138. In our study, the average areas of the granular cells were 541.7 ± 166.76 μm^2^ at 19 to 29 years, 674.66 ± 196.61 μm^2^ at 30 to 59 years and 648.98 ± 218.75 μm^2^ at 60 to 79 years; the average areas of the granular nucleus were 49.49 ± 14.87 μm^2^ at 19 to 29 years, 58.99 ± 12.99 μm^2^ at 30 to 59 years and 61.30 ± 20.92 μm^2^ at 60 to 79 years. The NC ratio of granular cells was 0.10 ± 0.01 at 19 to 29 years, 0.10 ± 0.02 at 30 to 59 years, and 0.11 ± 0.02 at 60 to 79 years. The cellular and the nuclear size of our results are much larger than that of Koehler's results, but the NC ratio of our results is smaller. Even though we do not know the source of the difference, one can check on previously published traditional histological images, as exampled in the epidermis image shown in page 465 of Ref. 32. In this image, one can find the average areas of the granular cells as 880.95 ± 128.26 μm^2^, which are close to our results. Combined with our previous study on the measurements of granular cells in Asian subjects 13, we concluded that the cellular and nuclear size of granular cells in Caucasian subjects changed with age with different trends while comparing with those observed in Asian subjects. The Caucasian subjects have on average a larger cellular size, nuclear size and NC ratio of granular cells, as compared to those in Asian subjects. The reason for these differences worth further investigations.

### 4.3 Comparison between results of average area of basal cells

**Figure 21:**
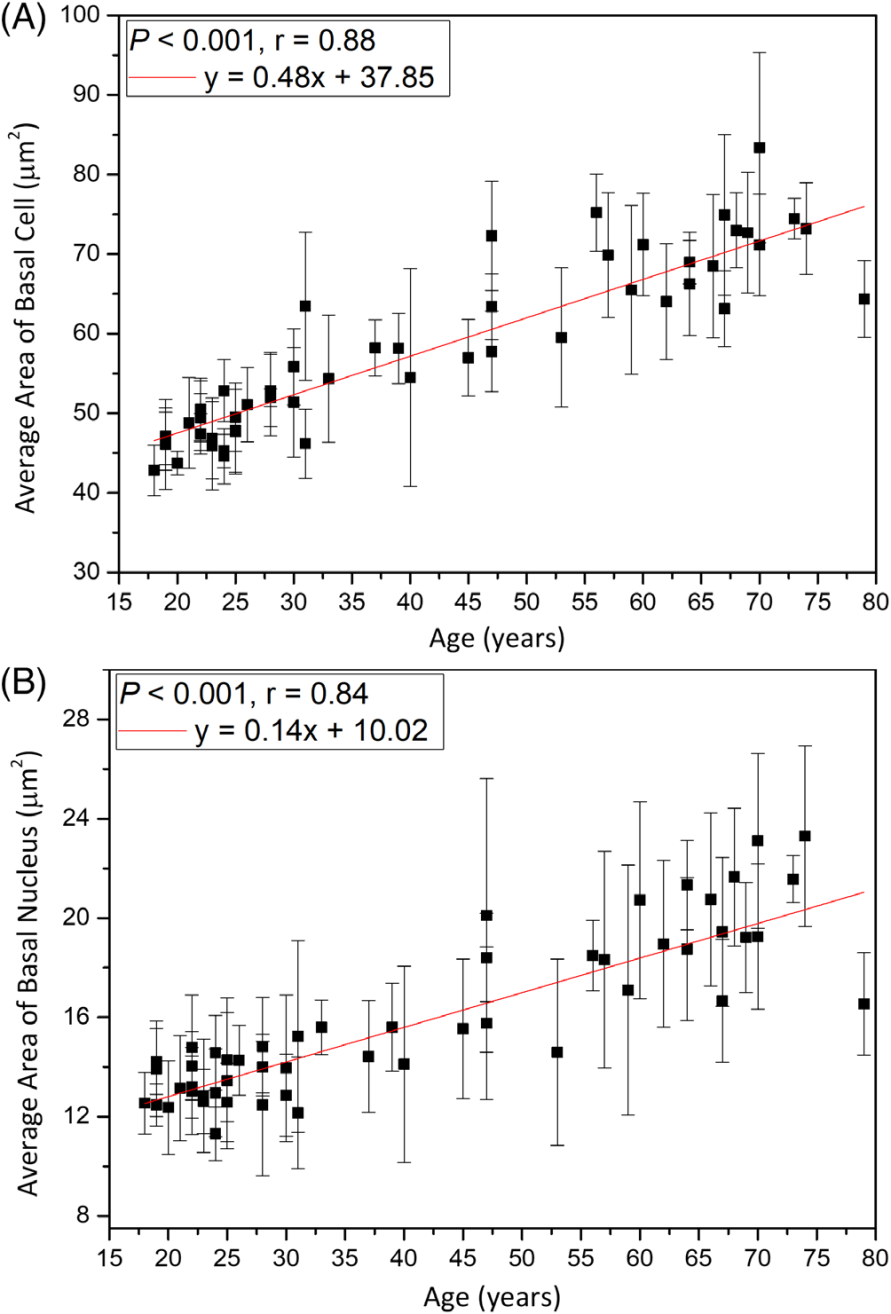
Average cellular and nuclear size of the basal cells vs age from the ventral forearms of Caucasian and Asian subjects (n = 52). A, In simple linear regression, *P* < .001. Correlation coefficient *r* = .88. B, *P* < .001. Correlation coefficient *r* = .84

The average area of the basal cell and its nucleus was found to increase with advancing age in Caucasian subjects. Consistent with our results, previous in vitro cell culture studies have also shown that keratinocyte senescence associated with intrinsic aging exhibited an increase in cell size 33. Cellular size has been reported to be a major factor of the clonogenic ability of keratinocytes that declines with intrinsic aging 34. According to our results, the NC ratio remained constant with advancing age, in agreement with a previous study 30. Based on the similar cellular and nuclear sizes of basal keratinocytes in Caucasian subjects as those in the Asian subjects' skin among the same age group 13, we concluded that the primary factor affecting the cellular and nuclear sizes of basal keratinocytes is age, rather than race. Combining the data of Caucasian and Asian subjects, simple linear regression analyses show a strong positive correlation between the cellular and nuclear size of basal cells and age (Figure 21). Our study suggests that the cellular and nuclear size of basal cells are sufficient to evaluate our skin's true age among Caucasian and Asian racial groups.

### 4.4 Comparison between results of depth of dermal papilla zone

Neerken et al 31 reported that the depth of the dermal papilla zone of the Caucasian volar forearm showed a considerable decrease with increasing age. Compared with the results of our previous study 14, we further found that the depth of the dermal papilla zone decreased with age in Caucasian subjects with a faster rate as compared with Asian.

### 4.5 Comparison between results of 3D interdigitation index

Based on biopsy results, Timár et al 35 demonstrated a significant decrease in the 2D interdigitation index in the older inner forearm skin, which was attributed to the disappearing interdigitation between the epidermis and the dermis in association with intrinsic aging. A similar age‐related decrease was also found in the analysis of 3D interdigitation index in our previous study on Asian 14. Furthermore, the disappearance of interdigitation could be confirmed by the result showing decreased DPZ depth of with advancing age. Compared with the results of Asian study 14, we found that the 3D interdigitation index in the Caucasian was on average higher than that in Asian.

### 4.6 Comparison between results of 2D collagen density in DP and collagen volume per unit area

Even though our study showed that both the collagen density in DPZ and the average collagen volume per unit area vary with race (Caucasian higher than Asian), neither of them showed significant correlation with age. If previous studies had correctly reported that the dermal collagen density decreased with age 36, the loss could primarily occur in the reticular dermis or could have instead resulted from photo‐aging 37. Our results were also different from a previous report 8, which showed that the skin of Asian subjects exhibited increased collagen content when compared with the skin of Caucasian subjects. However, our studies were specifically analyzed only in the DP region.

## 5 CONCLUSION

In this study, we investigated whether the cellular and subcellular structural changes obtained by the in vivo HGM system in the epidermis and dermis were sufficient to characterize the racial differences in aged skin. Through the study, we hope to obtained morphometric information which would assist to reveal the process of intrinsic skin aging.

Using the image stacks obtained from the volar forearm on the Asian and Caucasian subjects, 14 parameters were defined to assess the morphological changes in the viable epidermis and the dermal papillae in a three‐dimensional point of view. Among these parameters, nine were found to be related to age in Caucasian subjects, but only five parameters were found to be related to the interaction between age and race.

Regarding the interaction between age and race, significant differences were observed among these racial groups in the maximum thickness of the viable epidermis, the cellular and the nuclear size of granular cells and the depth and the volume per unit area at the DPZ. That is, the maximum thickness of the viable epidermis, the DP volume per unit area and the depth of the DPZ in Caucasian decreased at much faster rates compared with those in Asian. The reason for this significant difference between two racial groups may be that the skin of Asian subjects ages at a much slower rate 13, 14. We suggest that the primary factor causing a different aging outlook between the Caucasian and Asian is the DPZ‐related parameters, while no significant differences were observed between the two racial groups in the cellular structural changes in the stratum basale. Several studies have demonstrated that there is an age difference between wrinkles of Caucasian and Asian subjects, which suggests the importance of the wrinkle analysis as an evaluation criterion of skin age. Our study reveals that the cellular and nuclear areas of the basal cell increase with age with the same trend between two racial groups, making these parameters ideal to define intrinsic skin age. In this study, we also demonstrated that our proposed histological and epidermal/dermal parameters could serve as a benchmark to study racial differences of intrinsic aging while extraordinary spatial resolution can be provided by the in vivo slide‐free stain‐free HGM technique.

## AUTHOR BIOGRAPHIES

Please see Supporting Information online.

## Supporting information


**Appendix S1.** Supporting Information.
