# Supplementary material for: Comparative analysis of intrinsic skin aging between Caucasian and Asian subjects by slide‐free in vivo harmonic generation microscopy
Source: J Biophotonics. 2019 Dec 5;13(4):e201960063. doi: 10.1002/jbio.201960063 (PMC7894538; doi:10.1002/jbio.201960063)
Supplement: Supplementary file 1 — Appendix S1. Supporting Information. [file JBIO-13-e201960063-s001.docx]

**Kuan-Hung Lin** received his B.S. degree in Electronic Engineering from National Kaohsiung University of Science and Technology, Kaohsiung, Taiwan, in 2015, and his M.S. degree in Biomedical Electronics and Bioinformatics from National Taiwan University, Taipei, Taiwan, in 2018. His current research interests include biomedical electronics and medical image processing.

**Yi-Hua Liao** received her MD degree from the College of Medicine, National Taiwan University, and her PhD from the Graduate Institute of Pathology, College of Medicine, National Taiwan University, Taipei, Taiwan, in 1996 and 2007, respectively. She is now an Associate Professor in the Department of Dermatology, National Taiwan University, and a supervisor of the Taiwanese Dermatological Association. She is responsible for dermatologic surgery special clinic, cosmetic surgery and laser special clinic.

**Ming-Liang Wei** received his B.S. and M.S. degrees from National Taiwan University (NTU), Taipei, Taiwan. Currently, he serves as a research assistant at the NTU Molecular Imaging Center and works on studying the clinical application of HGM for skin cancer and biomedical imaging analysis.

**Chi-Kuang Sun** is a Life Distinguished Professor in the College of Electrical Engineering and Computer Science and the College of Medicine at National Taiwan University (NTU), Taiwan. He received his Ph.D. in Applied Physics from Harvard University in 1995 and currently serves as Chair of the Photonics Program, Ministry of Science and Technology, Taiwan. Chi-Kuang Sun’s research involves optical molecular imaging, nonlinear microscopy, ultrafast phenomena, nano-ultrasonics, THz health care, advanced femtosecond laser technologies, and applications in virtual biopsy diagnosis, treatment and therapy assessment, surgical guidance, wearable monitoring device, neural science, virus epidemic control, paleontology, interfacial water imaging, and boson peak studies. He leads the advancement and clinical applications of third harmonic generation microscopy for noninvasive differential diagnosis of skin lesions. Chi-Kuang Sun is a Fellow of OSA, IEEE, SPIE. He received the Outstanding Research Award from National Science Council (Taiwan) three times and the MERIT Award from National Health Research Institute (Taiwan) two times, the 2010 Engineering Medal from the Taiwan Photonic Society, the Leica Microsystems Innovation Award, the C.N. Yang Outstanding Young Researcher Award, the Academia Sinica Research Award for Junior Researchers, the Y. Z. Hsu Science Chair Professorship, the AmTRAN Chair Professorship, and the Academic Award from Ministry of Education, Taiwan. He served as the President of Taiwan Section of OSA and a Topical editor of Optics Letters.
